# Analysis of the testimonial evidence of Portugal’s first major forensic case: part II

**DOI:** 10.1080/20961790.2019.1682218

**Published:** 2019-11-07

**Authors:** Ricardo Jorge Dinis-Oliveira

**Affiliations:** a Department of Public Health and Forensic Sciences, and Medical Education, Faculty of Medicine, University of Porto, Porto, Portugal; b IINFACTS - Institute of Research and Advanced Training in Health Sciences and Technologies, Department of Sciences, University Institute of Health Sciences (IUCS), Advanced Polytechnic and University Cooperative (CESPU), CRL, Gandra, Portugal; c UCIBIO-REQUIMTE, Laboratory of Toxicology, Department of Biological Sciences, Faculty of Pharmacy, University of Porto, Porto, Portugal

**Keywords:** History of legal medicine, forensic sciences, forensic toxicology, Vicente Urbino de Freitas, Mário Guilherme Augusto de Sampaio, testimonial evidence, Flores Street

## Abstract

The crime possibly perpetrated by a doctor named Vicente Urbino de Freitas in 1890 is one of the most famous cases of poisoning, and it had echoes in the Portuguese and foreign press for several decades. This prestigious doctor was convicted of the fatal poisoning of his nephew. He also attempted the homicide of two nieces and their mother-in-law, who only escaped because they obstinately refused to comply with the “therapeutics” prescribed by the family doctor. The motive of the crime should have been Vicente Urbino de Freitas’ ambition to receive the family inheritance of his wife, the daughter of the well-known merchant José António Sampaio of Flores Street in Porto. Vicente Urbino de Freitas was convicted but doubt about his guilt persists for more than a century. This second work aimed to collect and analyse all the relevant and contradictory testimonial evidence of the prosecution and defence witnesses. This case represents an odd historical record obtained through more than 12 years of research on the first major significant Portuguese forensic case. Rare and unprecedented testimonial evidence and photographs were obtained from different countries and then repaired, since these also provide an important historical record of the medical photography.

## Introduction

**Figure 1.**
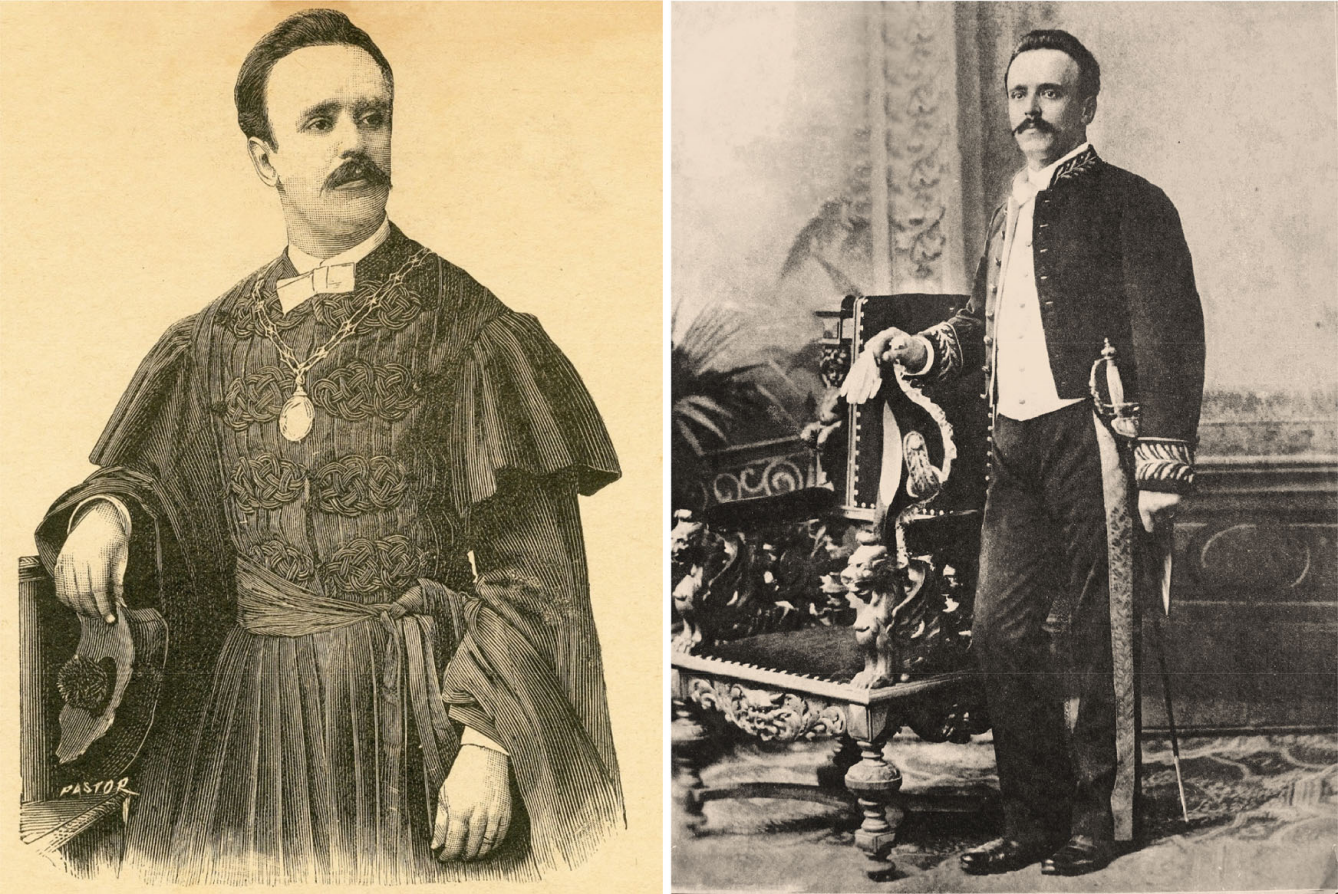
Recovered and restored portraits of Vicente Urbino de Freitas.

**Figure 2.**
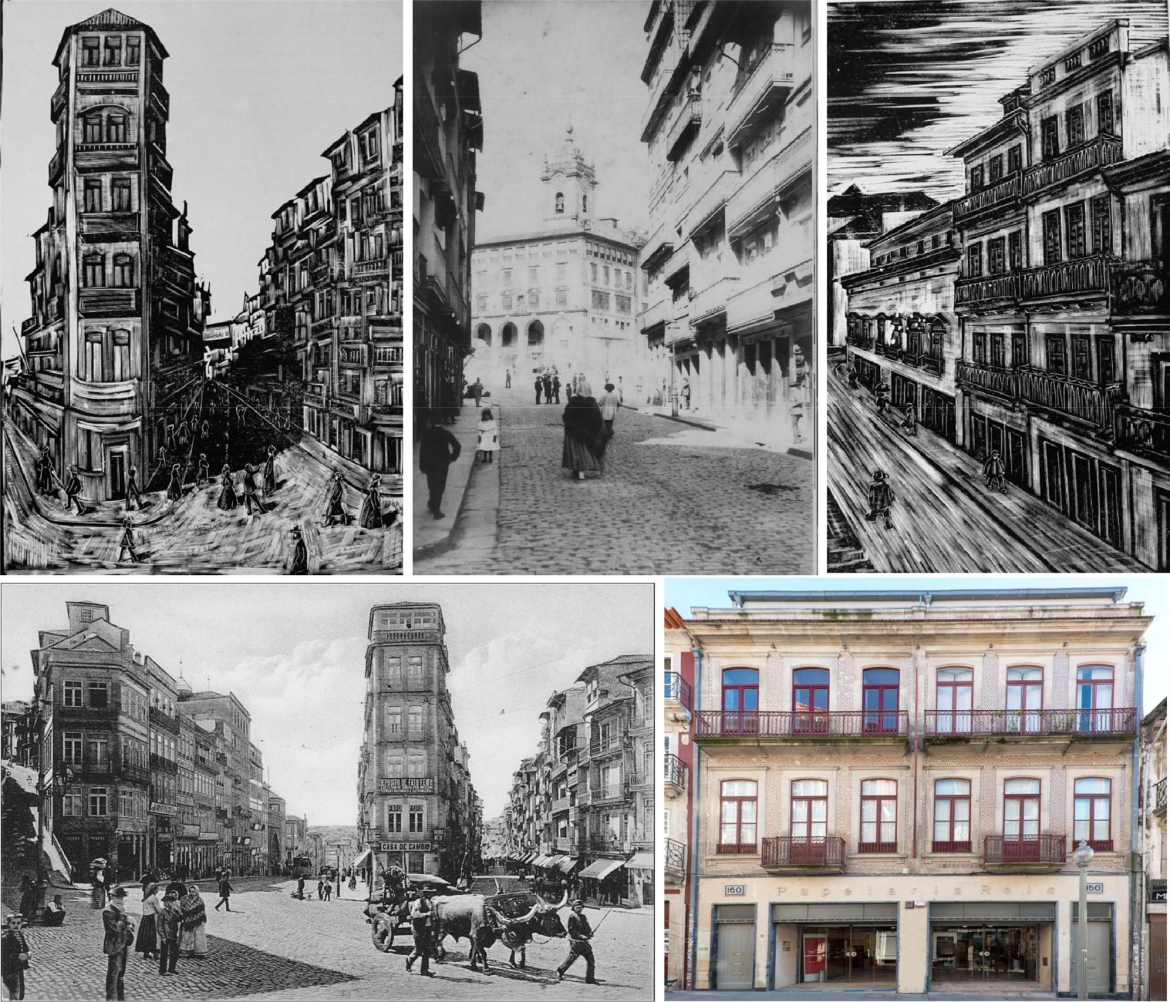
Photos and pictures of the Flores Street from 1890s. Vicente Urbino de Freitas was born in the house indicated by the n° 150–160. The Typography and Stationery stores of António Freitas Fortuna functioned in the ground floor.

**Figure 3.**
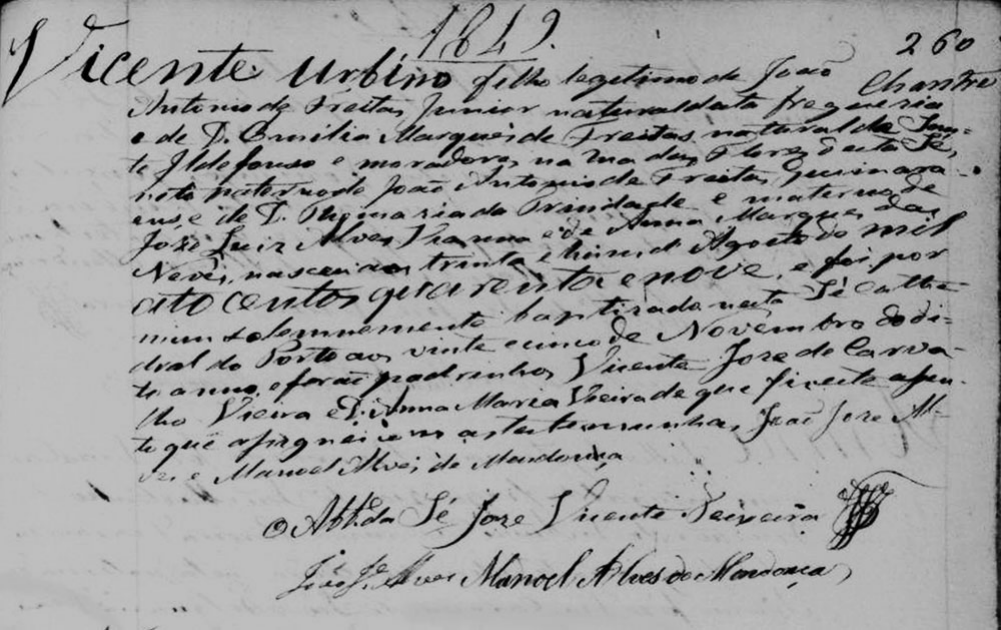
Recovered and restored baptism certificate of Vicente Urbino de Freitas in 1849.

In the vast gallery of infamous criminals that have been brought to justice, the poisonous Vicente Urbino de Freitas (Figure 1) is one of the most well-known [1,2]. Vicente Urbino de Freitas was born in Porto, at Sé Parish, Flores Street (formerly known as Santa Catarina das Flores Street) n° 150–160 on August 31, 1849 [3]. Flores Street was opened in 1518 by King Manuel I. Despite some decline at the beginning of the 20th century, Flores Street has become one of the most emblematic places in the Porto Historic Centre (Figure 2). The son of João António de Freitas Junior (a paper merchant from Flores Street) and Emília Marques Alves Viana, Vicente Urbino de Freitas was baptized in the Porto Cathedral on November 25, 1849 (Figure 3). After the death of his parents, his education was largely in the care of his brother, João António de Freitas Fortuna.

**Figure 4.**
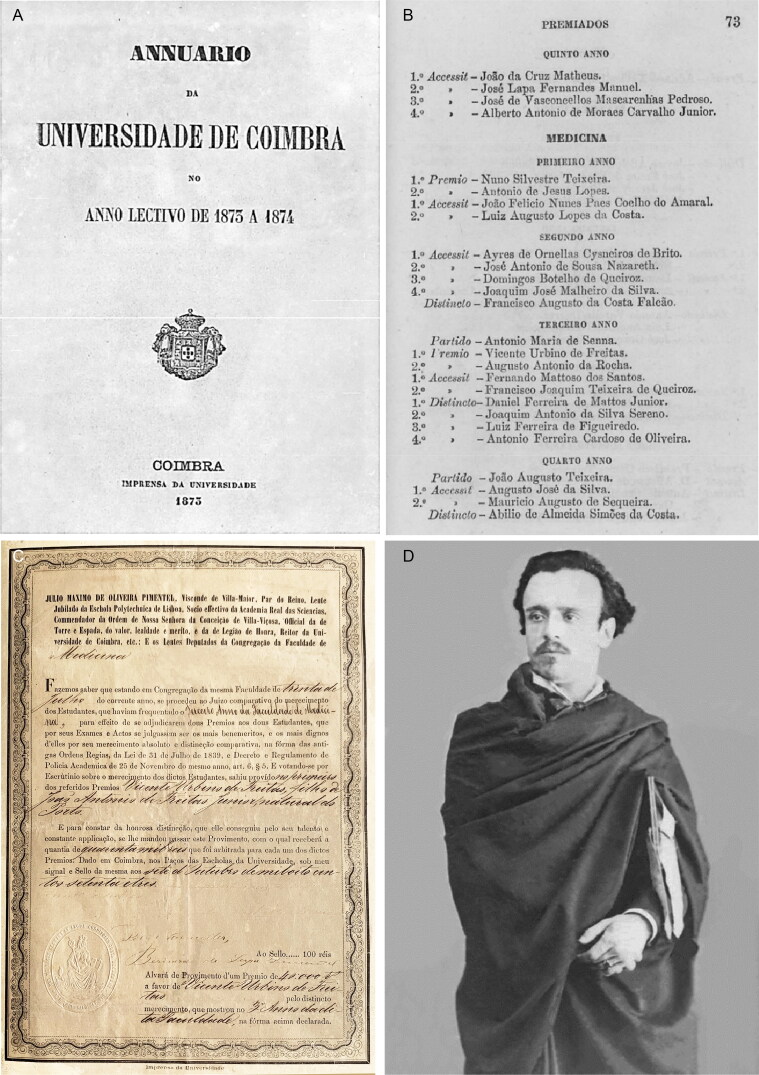
Directory book attesting that Vicente Urbino de Freitas received the best prize student in the 3rd academic year (A and B) and corresponding certificate (C). Portrait of Vicente Urbino de Freitas after completing the Medicine degree (D).

**Figure 5.**
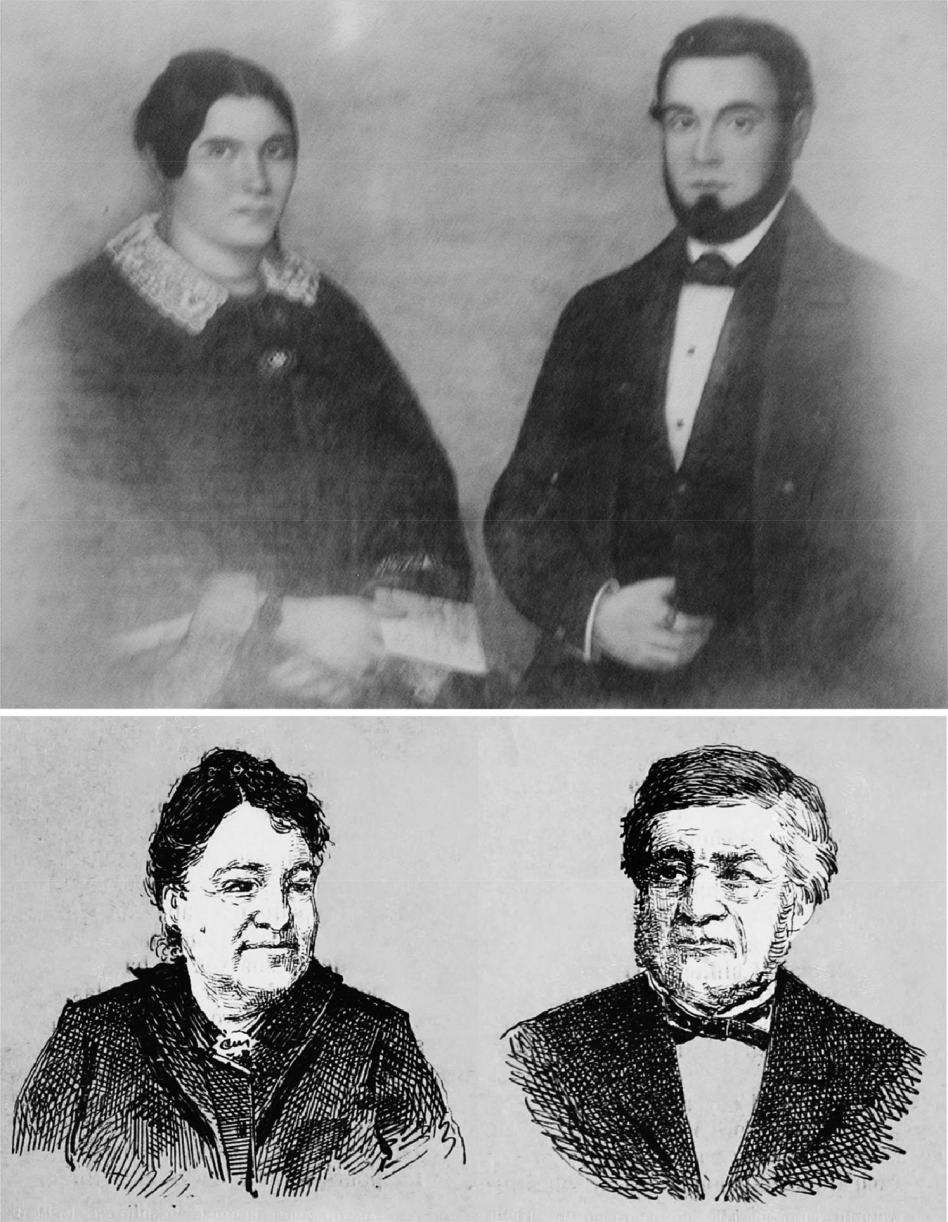
Recovered and restored portraits of Maria Carolina Bastos Sampaio and José António Sampaio.

**Figure 6.**
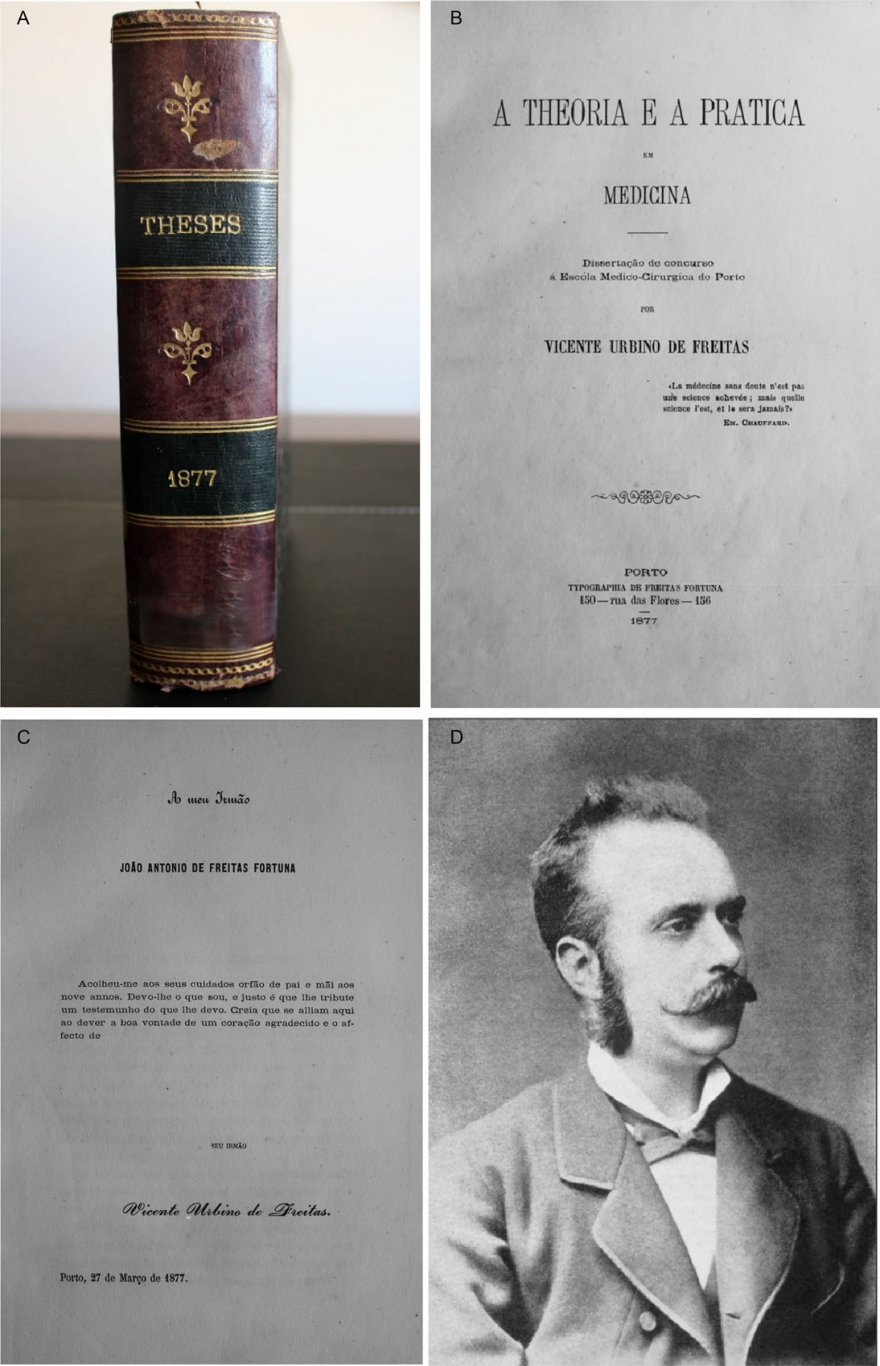
Recovered and restored Vicente Urbino de Freitas’ inaugural dissertation (thesis; A and B) dedicated to his brother, João António de Freitas Fortuna (C and D).

**Figure 7.**
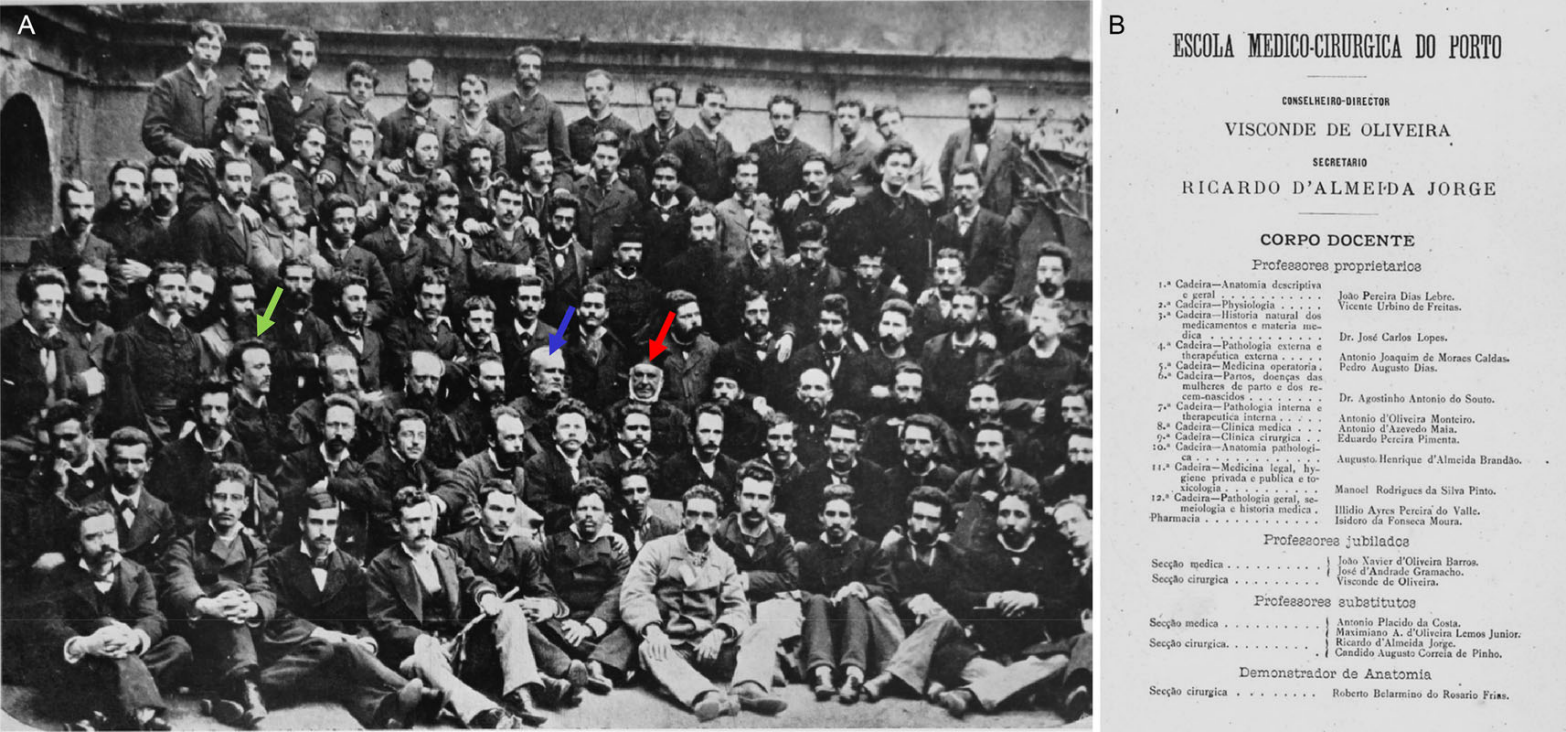
Porto Medical-Surgical School students and professors (A). Arrows shows Vicente Urbino de Freitas (green), the Director Viscount de Oliveira (purple) and José Frutuoso Aires de Gouveia Osório (red). List of professors of the Porto Medical-Surgical School of 1888 (B).

**Figure 8.**
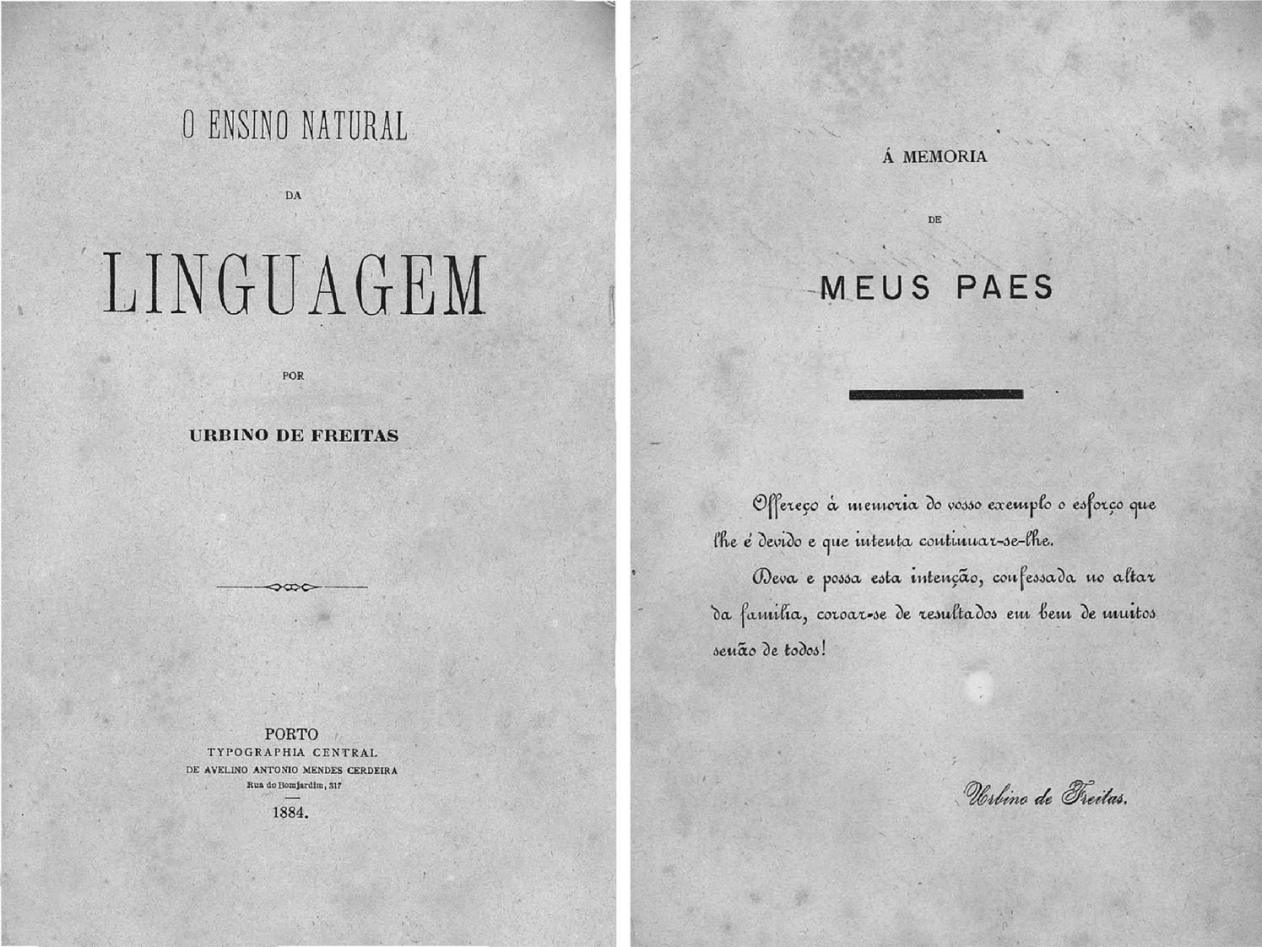
Recovered one of the most famous Vicente Urbino de Freitas’ scientific work dedicated to his parents, João António de Freitas Junior and Emília Marques Alves Viana.

**Figure 9.**
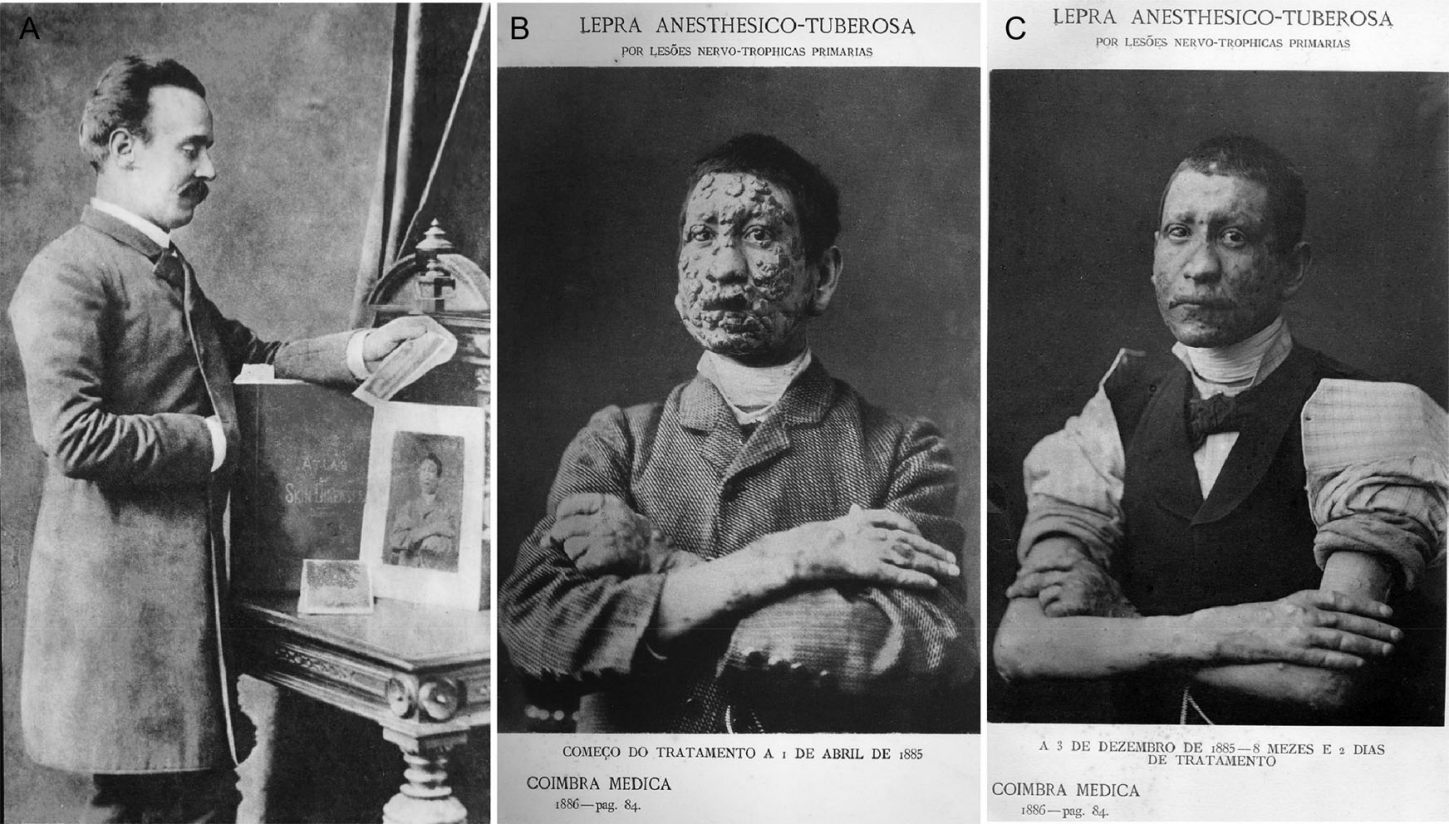
A leprosy patient (B and C) treated by Vicente Urbino de Freitas (A). The last two photos were a kind gift of the Dr. João Edward Clode and were provided by ​​Coimbra​ ​Médica: Revista​ Quinzenal de Medicina e Cirurgia (Medical Coimbra: Biweekly Journal of Medicine and Surgery)] in its edition of 6th year, nº6, march, 1886.

In 1875, Vicente Urbino de Freitas concluded a course in medicine at the Faculty of Medicine of the University of Coimbra with brilliance, winning several awards of merit throughout his studies and earning the classification of *nemine discrepante* (i.e. unanimously, Figure 4). In 1877, he married Maria das Dores Basto Sampaio Freitas, the daughter of José António Sampaio and Maria Carolina Basto Sampaio (Figure 5). On March 27 of that same year, he presented his dissertation entitled *Theory and Practice in Medicine* [4] to the Porto Medical-Surgical School (Figure 6A,B); the work was dedicated to his brother, João António de Freitas Fortuna (Figure 6C,D). Vicente Urbino de Freitas obtained a lecturer position, and by a decree on September 6, 1878 (from the Minister of the Kingdom the Marquis of Avila), he was named the substitute teacher of the Medical-Surgical School of Porto. In November 3, 1887, he accepted the responsibility for the 11th curricular unit of Hygiene and Legal Medicine [5], as a vacancy had arisen following the death of Professor José Frutuoso Aires de Gouveia Osório (1827–1887; Figure 7A). However, the 11th curricular unit was given to Professor Manuel Rodrigues da Silva Pinto, and Vicente Urbino de Freitas was placed in the 2nd curricular unit of Physiology (Figure 7B). Despite his stutter, his students’ reports stated that Vicente Urbino de Freitas’ lectures were clear. Some of his students became famous physicians as the psychiatrist Júlio de Matos and the ophthalmologist Edmundo Machado who diagnosed the incurable blindness of the writer Camilo Castelo Branco. One of his great works, entitled *The Natural Teaching of Language*, was dedicated to his parents (Figure 8) and explored and praised the reading method of his friend Cândido José Aires de Madureira (better known as “the Abbot of Arcozelo”) [6]. This work places the teaching of language as a social priority but respects the principles of anthropology in the definition of pedagogical laws by adapting to the stages of the natural development of the individual and avoiding dogmatic impositions that were doomed to failure. He was a noted physician, specializing in the treatment of leprosy (Figure 9), syphilis and morphea with several publications in the *Coimbra Médica* journal, some of them presenting the firsts collotypes in scientific articles in Portugal [7]. However, nothing made him as famous as the fact that he was convicted of the fatal poisoning of his nephew, Mário Guilherme Augusto de Sampaio, who died on April 2, 1890. The newspapers published daily details of the evolution of the forensic investigations, ranging from objective facts to rumours. This crime had incomparable international repercussions, even rivalling large judicial cases. The first major medicolegal case in Portugal both fascinated and stunned Portuguese society in the late 19th century [2]. This second work on the first major Portuguese medicolegal case aimed to collect and analyse all relevant and contradictory testimonial evidence from the prosecution and defence witnesses. This represents an odd historical record obtained through more than 12 years of research using books, newspapers, scientific journals and forensic reports scattered around the world. Of note, the manuscript also compiles a vast and outstanding assortment of photographs that depict not only the circumstances of the several intervenient of the case, but also of Vicente Urbino de Freitas’ life previous to the media hideous repercussions.

## Material and methods

The bibliographical research inherent to this reconstruction began in the middle of 2007 and was performed as previously described [2]. It entailed more than 12 years of research in libraries studying historical works, as well as the discoveries of the transcript of the 1893 criminal hearing and newspapers that had published various reports about the event. The research was not limited to literature in Portuguese (from Portugal and Brazil) but also included English and French documents, which is logical, given the worldwide coverage of this forensic case [8–16].

### The death of the brother-in-law José António de Sampaio Junior

**Figure 10.**
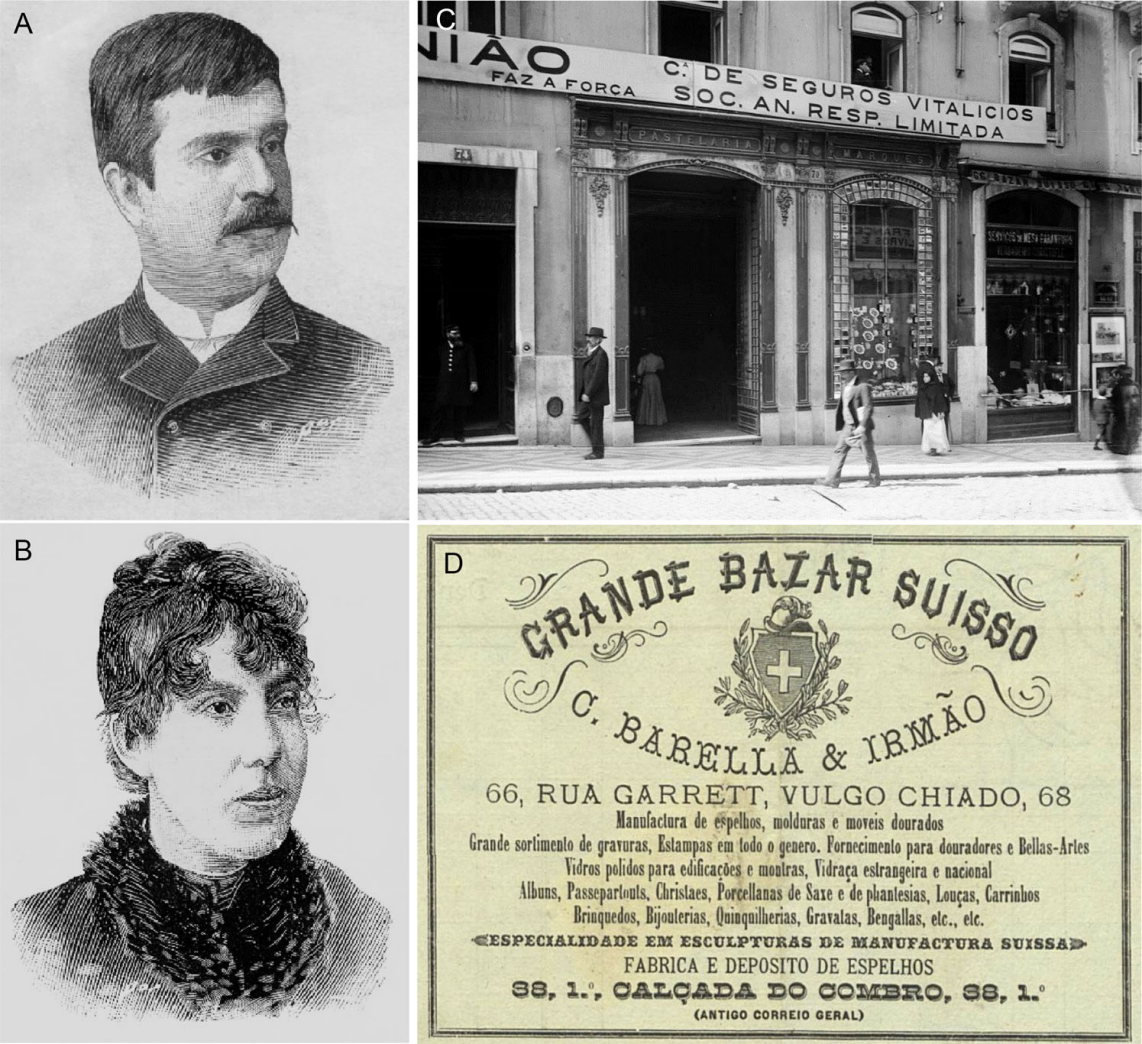
Recovered and restored portraits of José António de Sampaio Junior (A) and Lothie Karter (B), and the Suíço Bazaar (C and D) at Chiado of the brothers Celestino Barella and Albino Barella, where Lothie Karter worked.

José António de Sampaio Junior (Figure 10A), brother-in-law of Vicente Urbino de Freitas, following the death of his wife, Cacilda de Almeida Sampaio, became a well-to-do bohemian. He went to Lisbon, where he frequented the clubs and bars of the capital, which his father did not approve of. Whether it was the result of the life he led or a genetic predisposition, he often complained of stomach and liver pain. As mentioned by his new love, Miss Lothie Karter (Figure 10B), while in Lisbon in October 1889, he received a package in the mail from someone called “Motta” in Coimbra containing vials for maladies of the stomach and liver [11,13]. Lothie Karter was an English woman and a cashier at a Chiado store, “The Suiço Bazaar” located in n° 66–68 Garrett Street (Figure 10C,D), which was owned by the brothers Celestino Barella and Albino Barella. José António de Sampaio Junior was particularly surprised to have received the vials, since he did not recognize the sender [11,13]. He did not administer the remedy, a providential fact, because, as reported by Lothie Karter, José António de Sampaio Junior suspected the contents to be prussic acid, a potent poison [11,13].

**Figure 11.**
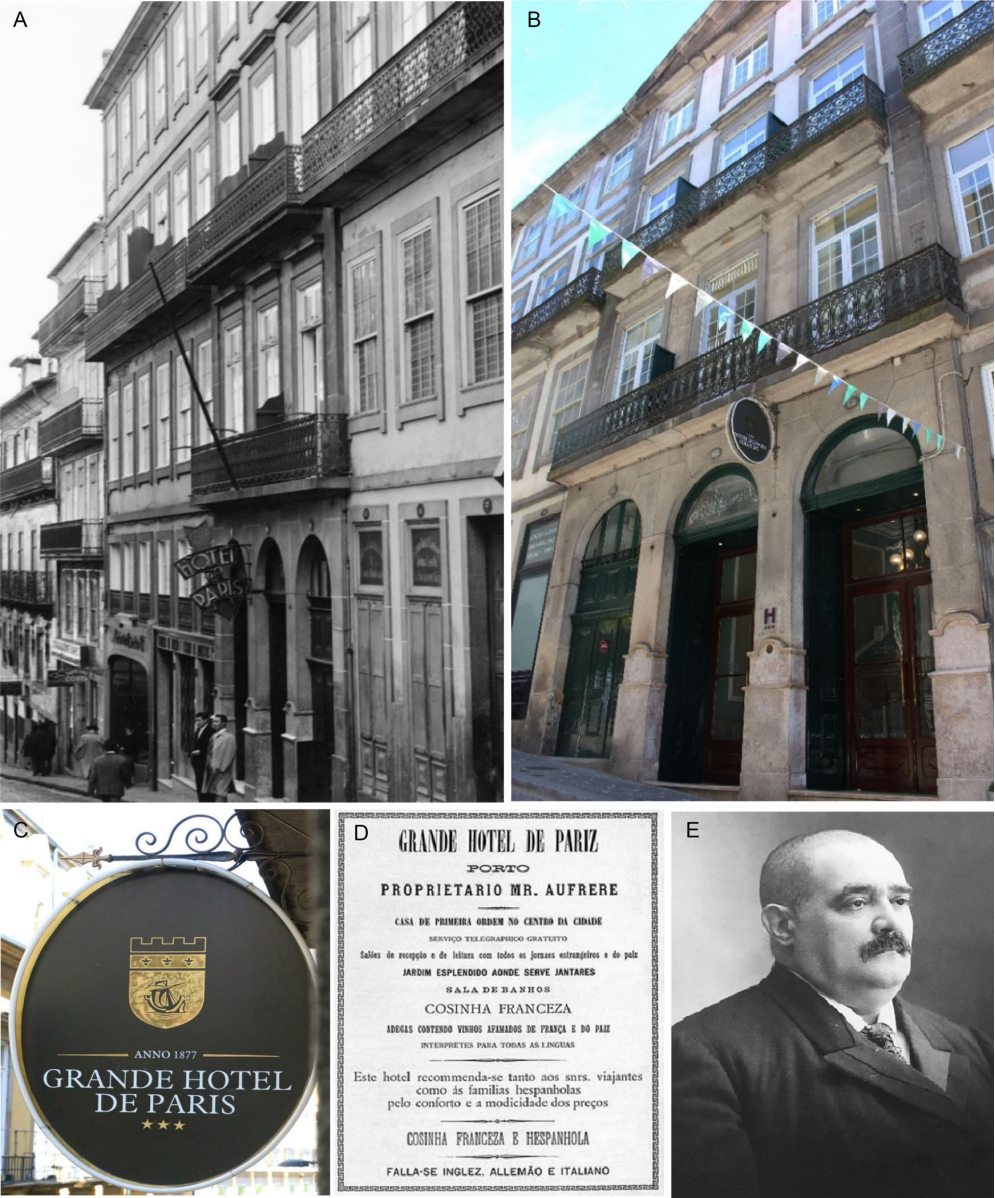
Grande Hotel de Paris of Porto façade from 1900s (A) and nowadays (B and C) and an advertise of banquets (D) directed by the French owner Joseph Aufrère (E).

On December 20, 1889, José António de Sampaio Junior left for Porto with Lothie Karter in an attempt to obtain his father’s blessing. They were installed in the Grande Hotel de Paris (located on Fábrica Street), the oldest hotel in the city, inaugurated in 1877, Mr. Joseph Aufrère being the director (Figure 11) [17]. On December 28, José António de Sampaio Junior visited and lunched with his brother-in-law, Vicente Urbino de Freitas, and returned to the hotel. He did not want to have dinner. On the 29th, he fell ill and suspected that it was due to a cold, so he dismissed his worries. The night passed well, and on the morning of December 30, he administered a purgative of magnesium citrate. Nevertheless, the illness progressed, and since “influenza” was a prevalent disease, after much insistence on the part of Lothie Karter, Vicente Urbino de Freitas was called.

Vicente Urbino de Freitas prescribed a hypodermic injection of pilocarpine (300 mg) that was bought in the Birra Pharmacy of Largo dos Loios. He personally offered to administer it to the patient’s left leg using a Pravaz syringe (i.e. a small hypodermic syringe with a hollow needle that made the first parenteral drug applications possible) of white metal and glass. After the administration, José António de Sampaio Junior, who had been sitting on the bed before, fell back into delirium, with cold sweat and shaking extremities, a heartbeat of 40 bpm, a temperature of 39 °C, miotic pupils and a loss of vision, partial apophenia, reduced mobility and sensitivity, and vomiting. Even in the face of these symptoms, Vicente Urbino de Freitas insisted on giving him a second injection, but he failed because the syringe was broken. Then, he came back with another instrument and was informed that his brother-in-law had alternated between fainting and having cold sweats. He replied, “This is nothing. With another injection, he will be good”, and proceeded to the second administration.

He then left, aiming to seek out the distinguished personal physician of the patient, Dr. José Carlos Godinho de Faria (born in Ceras, Alviobeira, Tomar), who had lived in Porto since 1871 and was a brother of the last Mayor of the municipality of Matosinhos in the transition from the Portuguese Monarchy to the First Republic, Dr. Francisco Fernando Godinho de Faria. A clinical report was provided, and Dr. José Carlos Godinho de Faria was invited to go to see José António de Sampaio Junior, since Vicente Urbino de Freitas argued that he did “not want to take responsibility for the treatment” of his brother-in-law. At approximately 2 am, the two doctors were at José António de Sampaio Junior’s bedside, and the patient was in a very serious condition. Dr. José Carlos Godinho de Faria raised the possibility of a suicide or criminal intoxication and suggested that José António de Sampaio Junior’s mother (who accompanied him as he died) and another doctor, Dr. Francisco José de Sousa Loureiro, be called.

The three doctors jointly prescribed the application of stimulants on the skin and the administration of purgative ointments and diagnosed cerebral congestion. On December 31, José António de Sampaio Junior recovered his vision, although his pupils were miotic, and he continued to improve until January 2, 1890 with the prescription of effervescent salts from his personal physician, who became the 15th prosecution witness. At 2 pm that day, Vicente Urbino de Freitas visited his brother-in-law and said he would need a new injection. Dr. José Carlos Godinho de Faria arrived shortly thereafter, and both agreed to the injection of 250 mg of caffeine citrate once it was obtained from the Birra Pharmacy. A servant named John Duran went to buy the prescribed formula and handed it to Vicente Urbino de Freitas.

The director of the hotel, Mr. Joseph Aufrère (Figure 11D), arrived by 1 pm and inquired him about the health of the guest he so esteemed. He also became a witness in the process because he was present in the last hours of José António de Sampaio Junior’s life. In his testimony, he mentioned that the prescription arrived at approximately 3 pm, and Lothie Karter stood between the two beds in the room while Maria Carolina Basto Sampaio sat on the couch [11,13,17]. Accompanying the two ladies was Vicente Urbino de Freitas. No one spoke, and Joseph Aufrère, respecting this silence, leaned over the back of the bed to better comfort the host.

Vicente Urbino de Freitas, after examining the vial brought from the pharmacy, poured the liquid into a chalice that was on a chest of drawers and went to the window, turning his back to those present (i.e. Maria Carolina Basto Sampaio, Lothie Karter and Joseph Aufrère). It was possible to observe him mixing the chalice and examining it in the light from the window, which was weak, given the narrowness of Fábrica Street [11,13,17]. He took a Pravaz syringe from a case and filled it with the liquid from the chalice, shaking it and observing it in the light of the window. He went back to the chest of drawers and emptied and refilled the syringe. He emptied the syringe once again, scrupulously wiped it, and put it into the case. He then poured the liquid from the chalice into the vial brought from the pharmacy, closed it and covered with a small paper cup with which the pharmacist had delivered the vial closed with a cork stopper.

Shortly, Dr. José Carlos Godinho de Faria entered the room and was informed the decline of the patient’s health status. He questioned Vicente Urbino de Freitas about whether it seemed appropriate to administer an injection. Agreeing, Vicente Urbino de Freitas suggested that Dr. José Carlos Godinho de Faria should perform the administration, and so it was agreed. In addition, after saying this, Vicente Urbino de Freitas took the syringe from the case and offered it to the other doctor together with the vial, which Dr. José Carlos Godinho de Faria examined. Vicente Urbino de Freitas said, “My colleague is very scrupulous!” [11]. Both physicians left but were called again later, since the patient’s health and the agony worsened. At the administration site, a “great black stain” developed [11]. The patient began vomiting again, violently and continuously, and his vomitus became bloody as he approached his death, which occurred at 6 pm on January 2, 1890. Vicente Urbino de Freitas strongly recommended that the vomitus be disposed of references [11,13].

On the last day, during one of the moments in which José António de Sampaio Junior appeared to be improving and had recovered from his aphonia, he told Lothie Karther, “I am persuaded that it was the injection of pilocarpine that made me feel bad…A thought passed by my head now…may God forgive me…even I cannot say to you…however, what I can say, is that my death is inevitable.” Near the last moments of his life, with his eyes glazed and his voice gone, he said to his mother, “I recommend you this poor girl. She is an angel.”

Joseph Aufrère questioned Vicente Urbino de Freitas about the cause of death of his brother-in-law, and the possibility of cerebral congestion was raised. Joseph Aufrère responded, “But I have already seen two men with this disease, my predecessor and my guest, Justino Moniz, and none of them had vomiting” [11,13]. Then, Vicente Urbino de Freitas elaborated a technical and medical explanation the hotelier was unable to understand. Joseph Aufrère lamented the death of the poor man, saying, “He died so young!” Vicente Urbino de Freitas replied, “He was a madman, a scoundrel, who shamed the family. Didn’t you notice the evidence of mental illness?… His whole family is like this. They all die by the same way” [11,13].

José António de Sampaio Junior was buried the next day, before law enforcement clarified his cause of death. This mysterious death in room 204 of the Grande Hotel de Paris has never been unravelled.

### The death of the nephew Mário Guilherme Augusto de Sampaio

**Figure 12.**
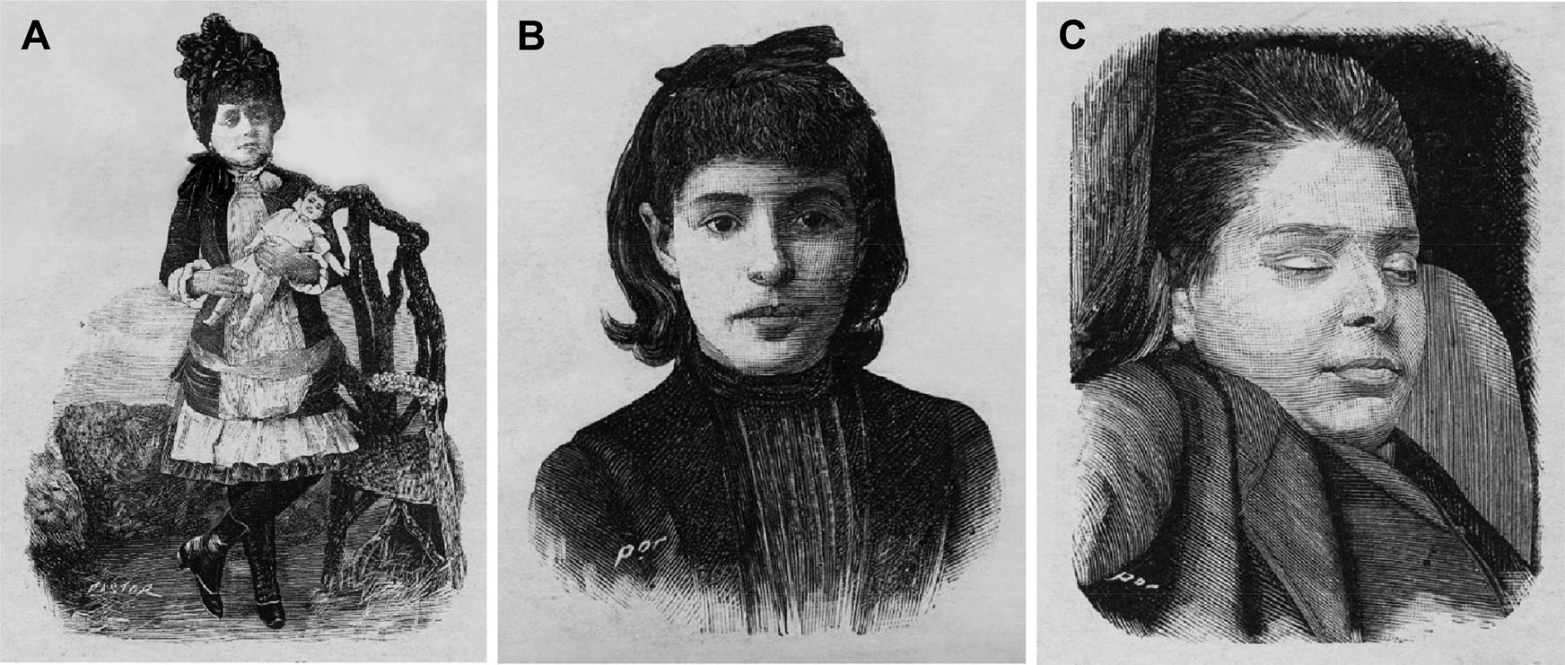
Recovered and restored portraits of Berta Fernanda Sampaio (A), Maria Augusta Sampaio (B) and Mário Guilherme Augusto de Sampaio (C).

Called to testify in court, Maria Carolina Basto Sampaio reported that the following events occurred in the house of Flores Street [8]: “On March 29, 1890, a package was sent to my house by Lúcio Artins, addressed to Berta Fernanda Sampaio (Figure 12A). Before leaving home, I recommended that no one open the package and they complied [2,11,13]. The next day, Sunday, after dinner, I showed the package containing almonds and chocolate cookies, and I distributed the almonds among my grandchildren. Vicente Urbino de Freitas showed up that afternoon. On March 31, everyone was in good health. At dinner, I distributed the chocolate cookies to the grandchildren at their request, and I also had some food. Berta Fernanda Sampaio and Maria Augusta Sampaio (Figure 12B) said the chocolate cookies were bitter, but Mário Guilherme Augusto de Sampaio (Figure 12C) ate the whole cake and there was still a bit left by Berta Fernanda Sampaio. I also found them to have a weird taste, but I dismissed it. The children went to play, and after some time, Maria Augusta Sampaio told me that Berta Fernanda Sampaio was very agonized. I gave them sugar water, which I asked the “negroid housekeeper”, Maria Luisa dos Anjos, to get, and I also gave them Eno^®^ fruit salts (to induce vomiting) that I had at home, and I also offered some to the housekeeper since she complained of abdominal pain. Maria Augusta Sampaio also administered olive oil prepared by the cook, Emília Rosa da Cunha. All of them vomited and had a good night, but the same did not happen to me because I spent the night distressed. The children woke up refreshed, and by the midday on the 1st of April, my son-in-law appeared, and I told him what happened. He recommended drinking coffee and lukewarm water. In the afternoon, he returned, and because I was still very anxious and ill-disposed, I suggested my son-in-law give me something to ease the affliction. He then asked, ‘For what?’ [and I responded] ‘If you do not want to, I will call another physician to prescribe a therapy.’ He then prescribed me an emetic, which was bought from the Gomes Pharmacy on Flores Street and it produced immediate relief. I suggested preserving the vomitus to be examined. Contrarily, Vicente Urbino de Freitas said, ‘Throw it out, it does not matter!’, and therefore I accomplished. Vicente Urbino de Freitas then examined the children and, while palpating their stomachs, asked, ‘Does it hurt?’ ‘No,’ they answered. My son-in-law then said in a tone that impressed me, ‘It sounds incredible!’, and nothing was prescribed [11,13]. At 9 pm on April 1, Vicente Urbino de Freitas came back and asked about the children’s health. While he was with Mario Guilherme Augusto de Sampaio, [the latter asked to show] my son-in-law… his stamps collection, [and Vicente Urbino de Freitas] inquired about how many were missing. Mario Guilherme Augusto de Sampaio told him that the ‘Rome’ stamps were missing. ‘I will bring them to you,’ said Vicente Urbino de Freitas, who frequently presented the child with stamps for his collection. Before the children went to bed, my son-in-law recommended giving them some clysters. ‘For what?’ I asked. It seemed that they were already completely rehabilitated and did not present any kind of complaints. Vicente Urbino de Freitas said that it would be enough to have some clysters of *Melissa officinalis* (i.e. lemon balm). I asked the housekeeper if there was lemon balm at home, and she said no. She went to buy it. During this period, Vicente Urbino de Freitas left the room and went to the kitchen to ask the cook to warm some water, according to the cook’s testimony. Then, he came back to the room and I saw he had a mug and a coffee maker. Berta Fernanda Sampaio told me that she saw the uncle throw something into the liquid. My son-in-law wanted to give the clysters with his hand, to which the children objected, so the housekeeper gave them to the three children. Vicente Urbino de Freitas recommended that they be quiet and suggested that he stay overnight at my house. I refused the offer, and he left at 1 am. Mário Guilherme Augusto de Sampaio kept the clysters, but the girls did not and quickly began to evacuate [their bowels]. On the next day, April 2, at 7 am, Vicente Urbino de Freitas was already back to my home, saying that since I was worried, he came to see if everything was well with me and the children. I had never seen him in my home so early. I told him that Mário Guilherme Augusto de Sampaio was very sick with complaints and that the girls presented headaches and drowsiness. Vicente Urbino de Freitas prescribed additional clysters and advised using warm water but not letting it boil. The girls, as soon as they heard their uncle giving orders, started screaming and refused additional clysters. I tried to persuade them that they would feel better. Vicente Urbino de Freitas advised the housekeeper to measure carefully with the syringe and to avoid losing any formula. He left for home, recommending that the children lay down for a little. Mário Guilherme Augusto de Sampaio fell asleep and did not evacuate. This fact may have allowed enough time for the effect to be manifested. At approximately 2 pm I heard a scream from Mário Guilherme Augusto de Sampaio, who had woken up and said, ‘Mom, mom, the clyster killed me, and I do not want to die! Mom I cannot see you…Do not worry, but I am very bad…The house is surrounding me, but I do not see anything.’ Maria Augusta Sampaio and Berta Fernanda Sampaio also complained of the same symptoms, namely, hearing loss and that the house was surrounding them! Vicente Urbino de Freitas was called again, and, upon entering, said, ‘These children have been poisoned.’ ‘And by whom?’ I asked. ‘I do not know!’ he replied [2,11,13,18].

**Figure 13.**
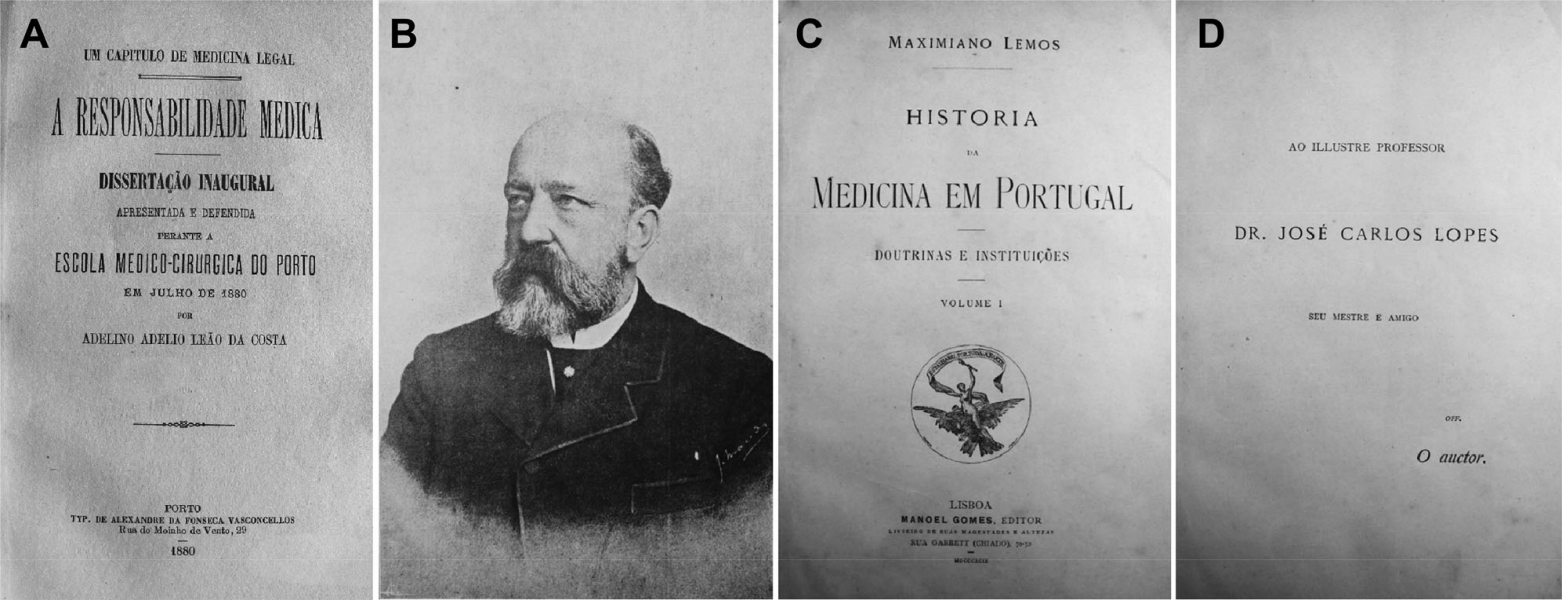
Recovered thesis of Adelino Adélio Leão da Costa about medical responsibility (A). Recovered and restored portrait of José Carlos Lopes (B) and by Maximiano Lemos in a book of the *History of Portugal Medicine* (C and D).

**Figure 14.**
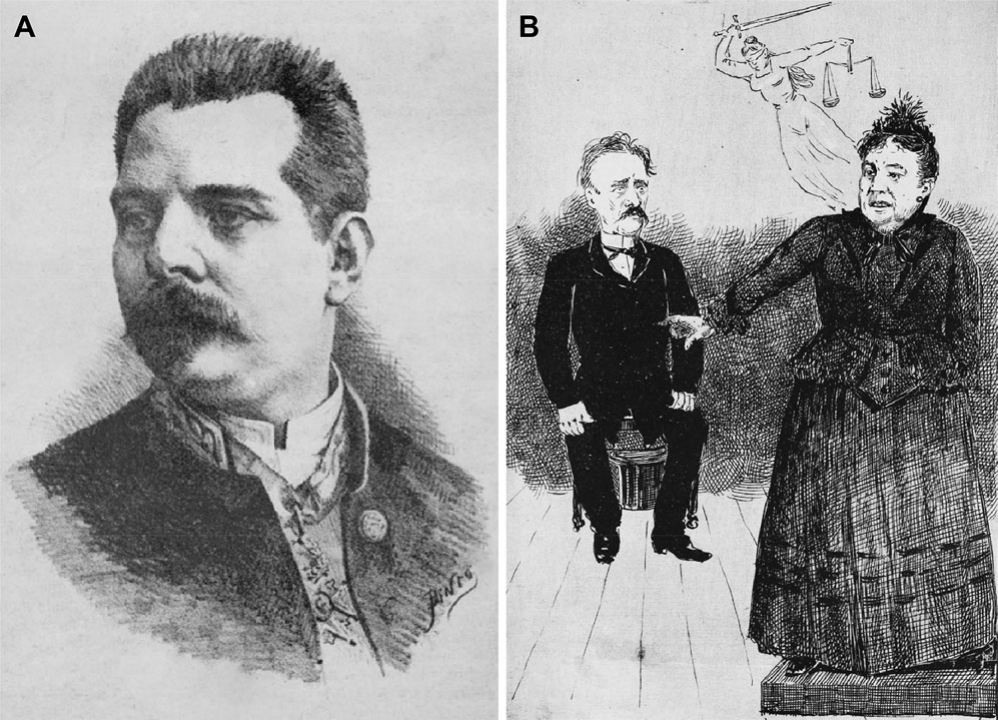
Recovered and restored portrait of Adriano Acácio Morais Carvalho published in the humorous and literary weekly *Maria Rita*, edition of December 10, 1885, under the artistic direction of Joaquim Maria Pinto and literary direction of Sá de Albergaria and António Cruz (A). Maria Carolina Bastos Sampaio’s solemn speech accusing Vicente Urbino de Freitas that deeply impressed the court (B).

Then Vicente Urbino de Freitas said that other doctors should be consulted. Dr. Adelino Adélio Leão da Costa came and then Dr. José Carlos Godinho de Faria, who arrived at 12 am. The former developed an interesting thesis about medical responsibility in 1880 (Figure 13A). Mário Guilherme Augusto de Sampaio was unconscious, with his teeth clenched in a convulsive crisis, exactly with the same symptoms exhibited by my dear son, José António Sampaio Junior. I remember that my son-in-law always refused to treat the people in the house, especially the children, with the argument that in case something happened to them, he did not want to be accused.” This account contrasted categorically with the solicitude and commitment of the time, namely, the treatment of the nephew and nieces, the administration of the clysters, and the offer to stay at the house on the night of April 1. The three children had severe symptoms, but those of Mário Guilherme Augusto de Sampaio were of exceptional gravity. Was it because he held onto the clysters longer and/or ate more candy? The truth is that after he screamed, he became aphonic and lost his senses, exhibited miosis and fell into a coma. As for the girls, the most relevant symptoms were vomiting, miosis, anuria, drowsiness, deafness, tinnitus, cold sweats and agony. Dr. José Carlos Godinho de Faria suggested consulting Dr. José Carlos Lopes (Figure 13B) in order to have assistance in identifying the toxic [11,13,18]. Indeed, he was the most competent professor of pharmacology and toxicology at the Medical-Surgical School in Porto and today has a room named after him in the History of Medicine Museum, according to Maximiano Lemos, who edited a book in his honour [19]. Dr. Henrique Antero de Sousa Maia was also consulted at approximately 3:30 pm on April 2, and the three doctors unanimously agreed in conference on the hypothesis of poisoning and quickly predicted the death of Mário Guilherme Augusto de Sampaio. Meanwhile, Vicente Urbino de Freitas left to look for the illustrious professor José Carlos Lopes at the Medical-Surgical School; José Carlos Lopes then accompanied him to the house at Flores Street at approximately 7 pm. Before, however, at 5 pm Dr. Joaquim José Ferreira, another distinguished doctor from Porto, had also been called, and when he arrived, he saw Mário Guilherme Augusto de Sampaio already deceased and said, “In your house, there has been a crime! Your grandchildren are poisoned.” After hearing the other doctors’ reports, Dr. Joaquim José Ferreira said that although the first symptoms could be attributed to the poisoned cakes, it was inexplicable and very serious that they recovered after the administration of laxatives of fruit salts but the symptoms reappeared again with such intensity, causing death of one of the children after many hours. In addition, he questioned whether they had undergone any additional medical treatment. He suspected opium and *Atropa belladona* and suggested that the police chief of Porto, Adriano Acácio de Morais Carvalho (Figure 14A) be called. Approximately 7 pm, Vicente Urbino de Freitas returned with Dr. José Carlos Lopes, whom he had met at the Carmo pharmacy. On the way, Vicente Urbino de Freitas had informed Dr. José Carlos Lopes about the case, attributing the poisoning to the chocolate cookies. Although he also mentioned that there had been a remission of symptoms between the first and last manifestations, he concealed the double application of the clysters [2,11,13,18].

The Porto General Police Commissioner arrived and determined that the investigation had already begun. He talked to Vicente Urbino de Freitas for some time and then left. Maria Carolina Basto Sampaio noted that, as a recommendation, Vicente Urbino de Freitas told her, “Caution with the General Police Commissioner, who is a Frenchman and a scoundrel; and you, never say that I prescribed…Say that I not only did not prescribe medicine, but also did not touch the children.”

The unfortunate Mário Guilherme Augusto de Sampaio passed away at 5 pm on April 2, exactly 3 months after the death of his father with his hands contorted and his corpse “turned greenish”. The girls were constantly very drowsy, with muscular weakness, deafness, cold sweats, dizziness and agony until April 5. They then began to improve, but in order to be completely restored, one had to undergo rigorous treatment for a month and a half and the other for three and a half months.

After this testimony, the mother-in-law of Vicente Urbino de Freitas stood up and turned to him, declaimed in a solemn tone that deeply impressed the court (Figure 14B), “I swear here before God and men that it was this man who killed my son, José, and my grandson, Mário! It was this man whom I gave money to go abroad to learn about poisons to kill my family! I swear, Your Honour (the judge), I swear. I could not imagine that my son-in-law would be able to do such a monstrosity because we were always his friends. He also intended to incriminate Carlos de Almeida, the maternal uncle of Berta Fernanda Sampaio, who lived in Lisbon. However, the handwriting expert examination acquitted him [20].”

### The police investigation

**Figure 15.**
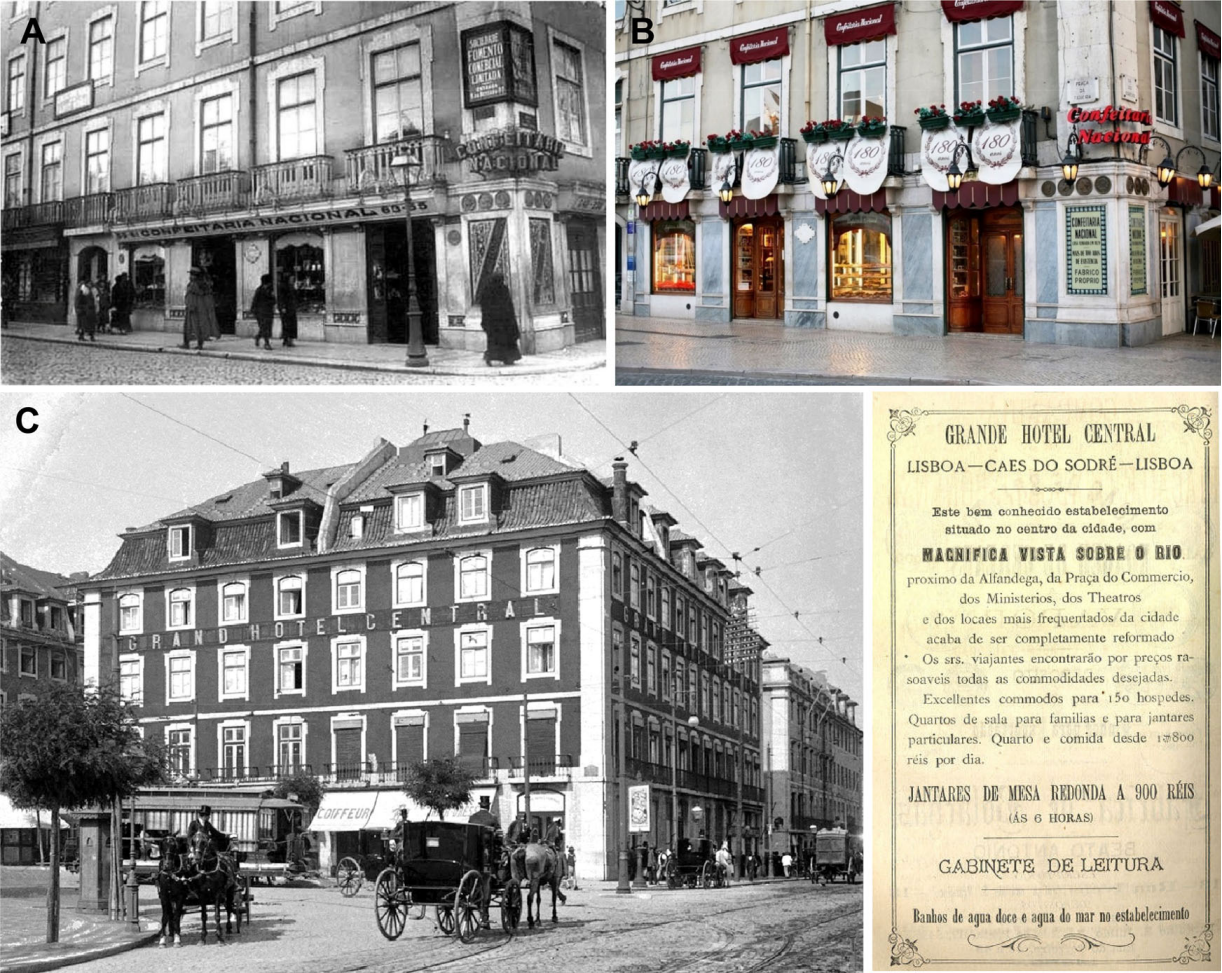
National Confectionery from 1890 s (A) and nowadays (B) where Vicente Urbinot de Freitas bought the chocolate and almonds and the Grande Hotel Central (C) of Lisbon.

Vicente Urbino de Freitas would have thought very long and hard about how to carry out the crimes in order to inherit the fortune of his in-laws. However, despite the lengthy planning, the final result was, in the end, highly compromising. From March 4 to 8, 1890 Vicente Urbino de Freitas was in Lisbon, where he bought a box of almonds and chocolate cookies that he brought to Porto. This was done in order to obtain a box with the mark of a confectionery from Lisbon to better support his plan. In Porto, he would have tried to add poison to the cookies. In the criminal investigation, it was also concluded that the box had been bought at the National Confectionery (Figure 15A), founded in 1829 by Baltazar Roiz Castanheiro. The National Confectionery is located at Betesga Street n° 59–65, a small street in the city of Lisbon that connects the Figueira and Rossio Squares and, interestingly, it was also the site of the first phone installations in Lisbon. Vicente Urbino de Freitas was recognized by one of the employees of the store.

Other police investigations demonstrated that Vicente Urbino de Freitas stayed in room n° 10 at the Grande Hotel Central (Figure 15C) at Remolares Square (presently Duque da Terceira Square) in Lisbon, on March 6 (he was seen at 1 am). It was also demonstrated that the package sent *via* Manuel Bento de Brito e Cunha was prepared in the stationery Gil (owned by Gil Carneiro) on Augusta Street, as declared by an employee named Augusto Pereira Mendes. The testimony of Augusto Pereira Mendes was reproduced in the *Jornal do Porto*, in its edition of 18 April of 1890. The Grande Hotel Central has an interesting history of being frequented by illustrious figures such as Júlio Verne and Eça de Queirós, inspiring the latter’s novels (e.g., *Os Maias*, *O Primo Basílio*). The Grande Hotel Central was considered the best hotel in Lisbon, a place where kings and presidents stayed. In the judicial proceedings, there is information that on March 6 in the morning, Vicente Urbino de Freitas asked the doorman at the Grande Hotel Central, Carlos Colombo, where he could buy good almonds for his fiancee [3,11,16]. The doorman said that Vicente Urbino de Freitas returned shortly afterwards with a package already addressed with the sender and receiver and asked him to mail it in a few days. Vicente Urbino de Freitas mentioned that he could not do so himself because he had to go to Mafra. He paid the bill and left. As mentioned above, Vicente Urbino de Freitas returned to Lisbon on March 7 and entered room n° 29 at 1 am. On March 8, he sought out the doorman, aiming to request the return of the package and stating that he had decided to personally deliver the almonds to his fiancee; then, he went back to Porto. Regarding his travels to Lisbon, Vicente Urbino de Freitas also told the Porto General Police Commissioner that he had come on the fast train on March 5, arriving at 12:30 am on the 6th of March and returned at 3:30 pm on the same day. The existence of accomplices in the crime was considered and investigated but was dismissed for lack of evidence [3,11,16].

### The crushing testimony of Manuel Bento de Brito e Cunha

**Figure 16.**
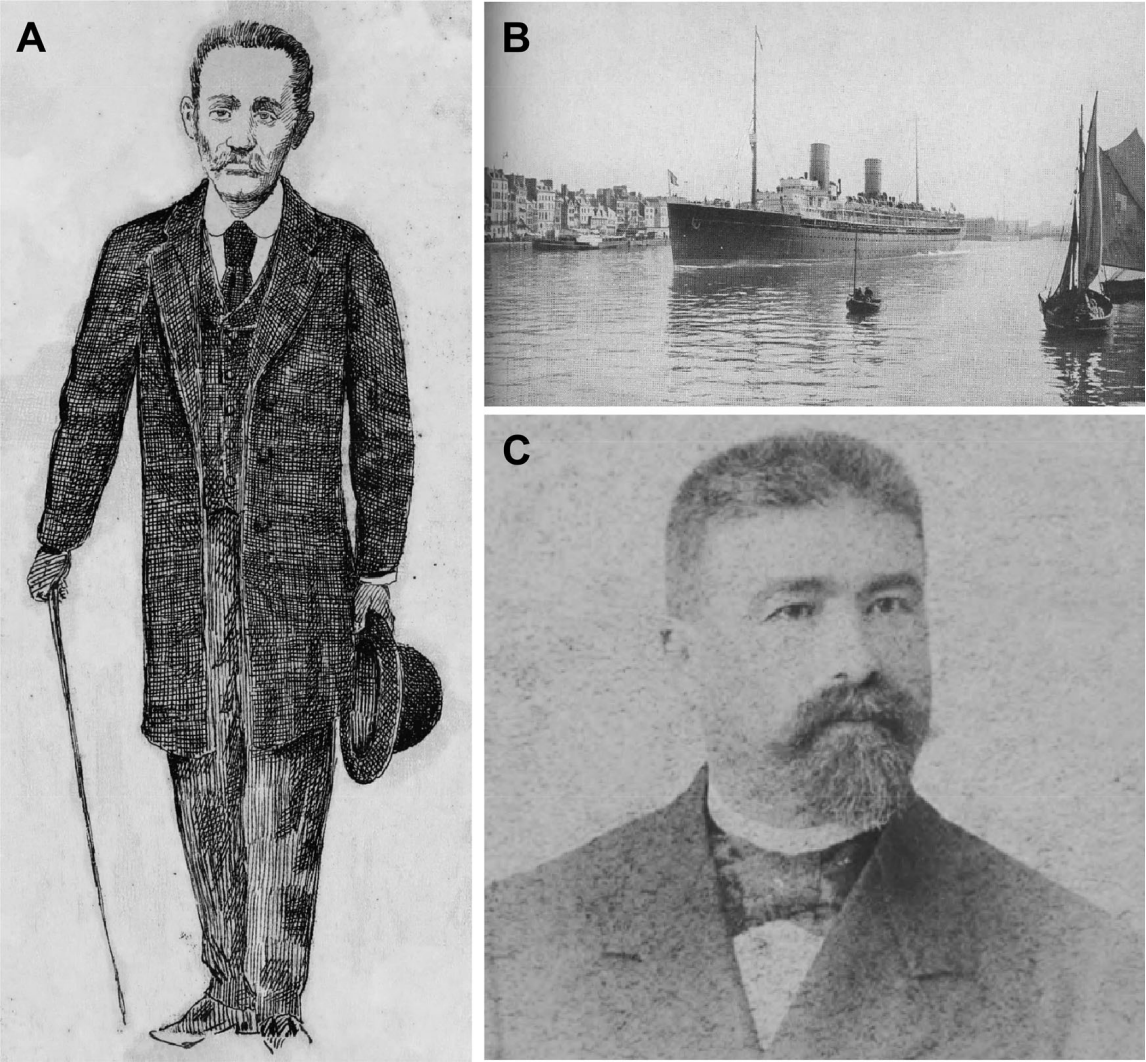
Recovered and restored portraits of Manuel Bento de Brito e Cunha (A), Daniel Luís Vieira de Abreu (C) and the French transatlantic vapor ship “Savoie” (B).

However, the question remained: accepting that these almonds bought at the beginning of March were the same ones sent on March 28, who had made the envelope and who would have sent the package from Lisbon? Moreover, as was proven, Vicente Urbino de Freitas was not in Lisbon on March 28. Indeed, although he made a train journey to Lisbon in the night of March 27, he then left the train in Coimbra. During this travel, he met Manuel Bento de Brito e Cunha (Figure 16A) in the train carriage; the latter was a merchant from Arcos de Valdevez who was about to embark on the French transatlantic steamboat “Savoie” (Figure 16B) from Lisbon, destined for Rio de Janeiro, Brazil [21]. Curiously, Daniel Luís Vieira de Abreu (1878—1902; Figure 16C) the son’s founder of the great Abreu Travel Agency, was the 11th witness to testify, as he was the one who had sold Vicente Urbino de Freitas the tickets. The Abreu Travel Agency, founded in 1840 in the city of Porto by Bernardo Luís Vieira de Abreu, was the first travel agency in the world to open to the public, and the agency still exists today and is owned by the founder’s descendants [21].

Vicente Urbino de Freitas, who presented himself as Eduardo Motta, had the art to convince Manuel Bento de Brito e Cunha to mail the package addressed to Berta Sampaio from Lisbon. Manuel Bento de Brito e Cunha, without any suspicion, took the package and enough money to pay the courier costs, and went to the capital. Vicente Urbino de Freitas left in Coimbra and waited for the new train that would bring him back to Porto. Therefore, Vicente Urbino de Freitas did not miss his duties at the Medico-Surgical School on March 27 and 28 and gave his class on the 28th at the scheduled time; at night, he attended a student recital at the São João Theatre. Months later, Manuel Bento Brito e Cunha, already in Brazil, became aware of the facts through the newspapers, but kept silent, fearing that he would be considered an accomplice of the crimes, even if his part was unknowingly done. Moreover, since he was in poor health, he was afraid to be called to Portugal to testify. Manuel Bento Brito e Cunha described what had happened in a letter addressed to his brother-in-law in Amares, Manuel José Martins Tinoco, who kept the former’s secret [1,3,11,13]. Due to health problems, Manuel Bento Brito e Cunha returned to Portugal to his home in Arcos de Valdevez. After some family disputes, Manuel José Martins Tinoco broke his silence and told his brother-in-law’s secret to a fellow goldsmith on Flores Street, Bento Augusto da Costa Guimarães, who, in turn, denounced him to the justice and became the 8th witness. Accompanied by Vicente de Urbino de Freitas, the police soon went to Manuel Bento Brito e Cunha’s house in Arcos de Valdevez, and Manuel Bento Brito e Cunha quickly recognized the defendant. His wife, Augusta Borges Nogueira e Cunha, also recognized the defendant. Manuel Bento Brito e Cunha also added in his testimony that the person on the train, whom he recognized as Vicente Urbino de Freitas, wore sunglasses, had his hat tilted over his eyes and had his coat collar turned upwards. In addition, Manuel Bento Brito e Cunha mentioned that this strange man irritated his wife.

### Other crushing charges

**Figure 17.**
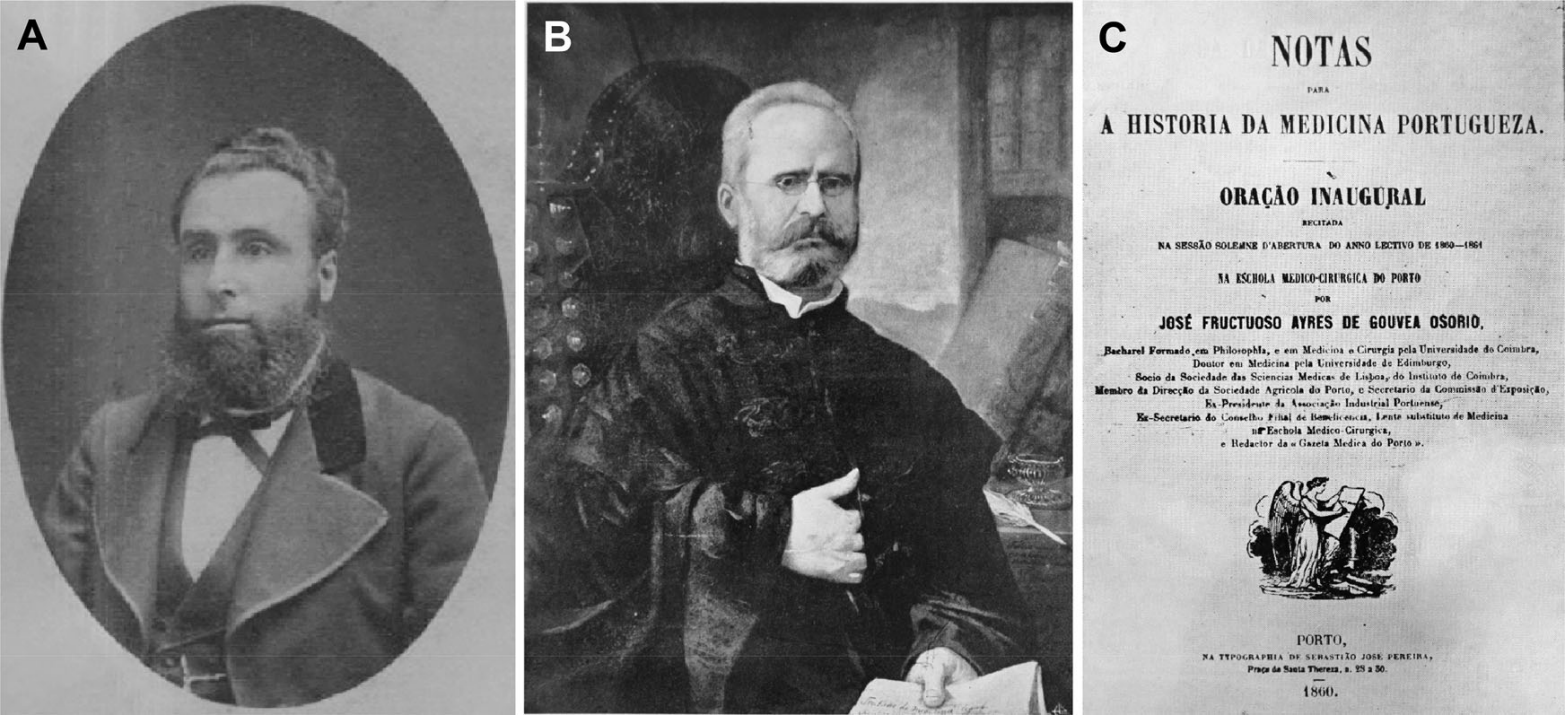
Recovered and restored portraits of José Ignacio Ferreira Roriz (A), José Frutuoso Aires de Gouveia Osório (B) and his first lecture in the opening ceremony of the academic year 1860/1861 (C).

If the testimony of Vicente de Urbino de Freitas’ mother-in-law was overwhelming, that of the 24th prosecution witness, João Augusto de Novais Vieira (known as “Novais da Madalena”; a public works employee), was not far behind. Regarding the poisoning of the Mario Guilherme Augusto de Sampaio, João Augusto de Novais Vieira said that he was only aware of the news because of the newspapers. Nevertheless, he also said that the idea of poisoning had come from the defendant’s grandparents. He mentioned that Vicente Urbino de Freitas' father had told him that his mother (i.e. the paternal grandmother of the defendant), who lived in Flores’ Street, had wanted to poison her husband through poisoned food. In addition, Vicente Urbino de Freitas’ father himself had already been a victim of poisoning through soup, a crime perpetrated by his his older sons when Vicente Urbino de Freitas was 9 years old [1,3,11,13]. The witness also spoke of the death of the famous banker, José Ignacio Ferreira Roriz (Figure 17A) on July 19, 1885, whose doctor was Vicente Urbino de Freitas. João Augusto de Novais Vieira accused the defendant of poisoning José Ignacio Ferreira Roriz, allegedly due to the latter’s financial issues with João António de Freitas Fortuna. José Ignacio Ferreira Roriz owned a bank on Flores Street (n° 1–3) that went bankrupt in 1876. Curiously, the banker was arrested and housed in the Porto Cadeia da Relacão prison, in cell n° 12 (known as the São João Room), which the writer Camilo Castelo Branco had previously occupied.

João Augusto de Novais Vieira also introduced other suspicions, namely, the implication of Vicente Urbino de Freitas in the death of Dr. José Frutuoso Aires de Gouveia Osório (Figure 17B), his protector and friend. Specifically, he declared, “I was not sure if he had been poisoned, but I heard that the defendant had invited him to dinner, and then he suddenly died while at home alone the morning after (August 23, 1887)”. I have no margin to speculate if Vicente Urbino de Freitas was involved in that death; this is solely based on circumstantial evidence. Nevertheless, it is somewhat bizarre and odd that it was Vicente Urbino de Freitas who carried the coffin during the funeral procession on the way to the burial in grave n° 214 in the 4th section on the private part of the Ordem do Carmo in the Agramonte Cemetery [22]. Vicente Urbino de Freitas was also implicated in the death of his daughter, but regarding this topic, practically nothing is known. In other words, the defendant was supposedly killing one person after another, particularly looking to extinguish the Sampaio family.

### The testimony of Vicente Urbino de Freitas to the police commissioner and in court

On the day of the trial, the court of São João Novo (a former convent) was filled, and for almost everyone, Vicente Urbino de Freitas was guilty, even before the trial began. The Portuguese newspaper *A República* wrote in its April 22, 1890 edition that “the man, until he proves the opposite, he is the most hateful poisoner”. Vicente Urbino de Freitas, 44 years old, was questioned by the judge in a session on November 30, 1890. “Stand up, the defendant”, said the judge. Vicente Urbino de Freitas rose, and there was absolute silence in the room, excepting in one corner of the room, dressed in black, his wife sobbed continuously. “Do you agree that there was poisoning?” the judge asked. “Absolutely. I supposed there was poisoning, but there was no crime”, Vicente Urbino de Freitas responded. The judge then asked, “What about the references made by your mother-in-law?” to which Vicente Urbino de Freitas responded, “I do not respond to my mother-in-law’s insinuations. I just want to mention that everything she and the housekeeper said is false”. Vicente Urbino de Freitas also mentioned that he had applied the lemon balm clysters to the children as suggested by his mother-in-law. Until the end of the interrogation, which was long and skillfully conducted by the judge, Vicente Urbino de Freitas denied everything that he was accused of references [1,3,11,13].

Vicente Urbino de Freitas tried to explain his trip to Lisbon on the grounds that he had gone to meet a married lady and stayed in Coimbra after missing the train at station because of intestinal discomfort. In Coimbra, he tried to get into closer contact with people who could corroborate and attest his presence in this city. Vicente Urbino de Freitas repeatedly justified the events with scientific facts, which the judge did not allow to continue. Therefore, the defendant, at one point, raised his hands to plead, saying, “Judge, my lord! For God’s sake, let me defend myself”. The judge replied, “Well, defend yourself, but answer my questions with precision and clarity and without embarking into the field of science, where I cannot follow you [11]”.

**Figure 18.**
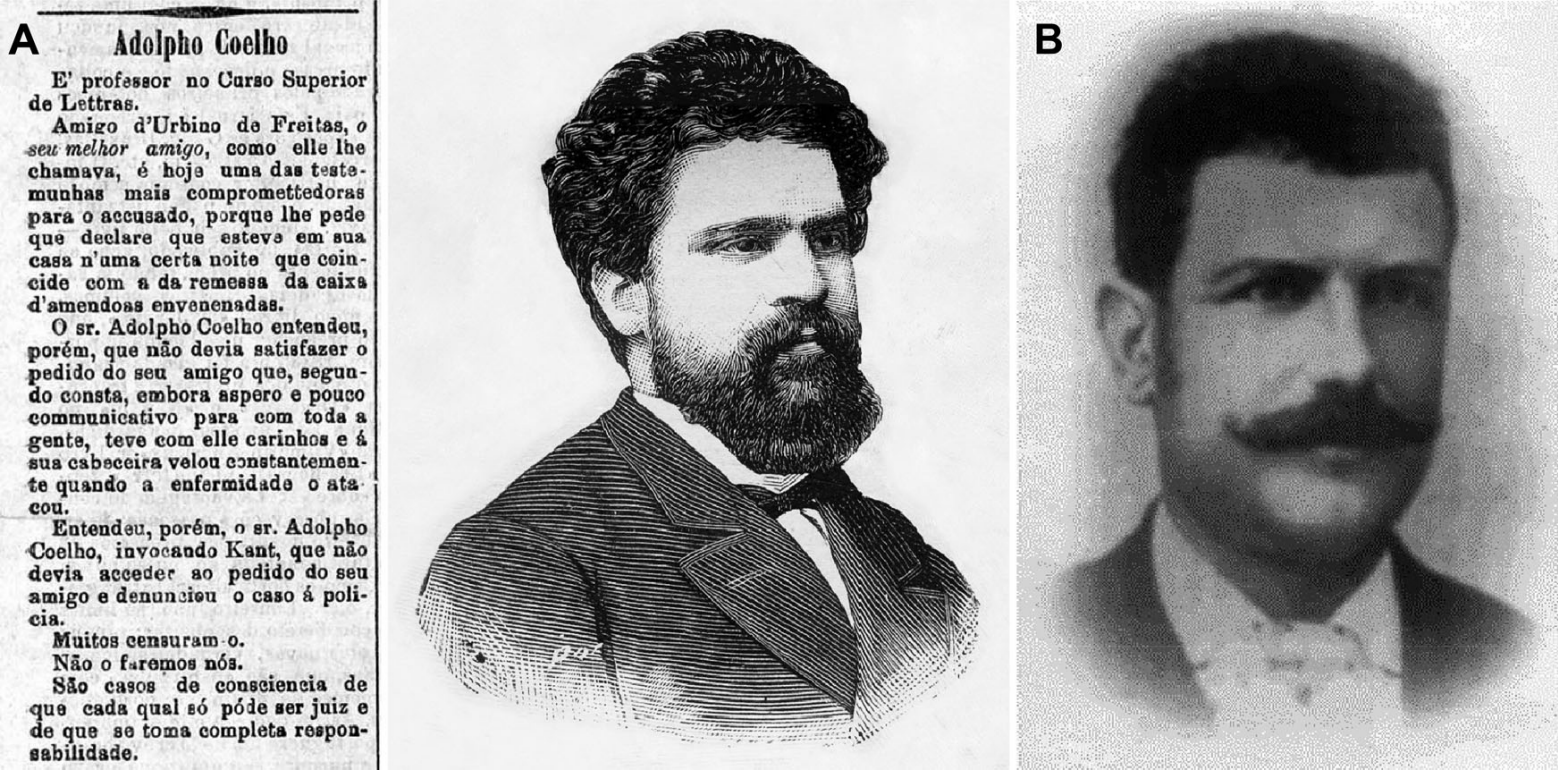
Recovered and restored portraits of Professor Francisco Adolfo Coelho and a copy of his statement (A) and Caetano Marques de Oliveira (B).

Regarding his travels to Lisbon, Vicente Urbino de Freitas told the Porto Police Commissioner that he had come on the fast train on March 5, arriving at 12:30 am on the 6th and returning at 3:30 pm on the same day. He returned to Lisbon on the 7th and left on the 8th at the same hour as the previous trip. On both occasions, he had dealt with translation matters regarding work about leprosy with his friend, Francisco Adolfo Coelho (1847–1919; Figure 18A). Francisco Adolfo Coelho was renowned professor of the Superior Course of Letters that lived in Lisbon at Belavista Lane at Lapa n° 9-1° floor. He was one of the most prominent Portuguese figures in early ethnographic and anthropologic study from the end of the 19th century to the first decade of the 20th century. On March 27, the day before mailing the package, Vicente Urbino de Freitas intended to go to the capital for the same reasons, but due to his abdominal discomfort, he left in Coimbra and then missed the train that followed to Lisbon. He stayed in Coimbra city until the 28th, catching the train back to Porto and arriving at 7:30 am. Francisco Adolfo Coelho not only denied the presence and hosting of his colleague, Vicente Urbino de Freitas, at his house on the days indicated but also declared that Vicente Urbino de Freitas had written a letter asking him that if he were questioned by the police to confirm Vicente Urbino de Freitas was present there to work on the book on leprosy. Francisco Adolfo Coelho mentioned that in addition to this letter, Vicente Urbino de Freitas had written others (four in total) and asked that Francisco Adolfo Coelho tear them all apart. Nevertheless, Francisco Adolfo Coelho did not do this; he saved and shared them with the public.

In the newspapers, the great ingratitude of Professor Francisco Adolfo Coelho was the subject of much debate. Indeed, when Francisco Adolfo Coelho suffered from heart disease and communicated his concerns to Vicente Urbino de Freitas, the latter solicitously offered that the former visit him at his house in Porto for treatment, and Francisco Adolfo Coelho was there for months, receiving affectionate dedication and medical care. When confronted with his friend’s statements and the letters he had written to him, Vicente Urbino de Freitas showed signs of despair and enormous sadness and said, “This looks incredible! Adolfo! My close and dear friend! It looks incredible!” Vicente Urbino de Freitas then told another version in which he came to Lisbon to meet a married woman, a patient from his Porto clinic. Nevertheless, she was never discovered.

Many other police investigations were carried out in Coimbra, Lisbon, and other regions of the country, but most of them added nothing more than entropy and delay to the judicial decisions. One investigation does deserve to be mentioned. In fact, Vicente Urbino de Freitas was not in Lisbon on March 27, since he was in Coimbra in the company of the distinguished physician, Dr. Caetano Marques de Oliveira (1864–1926; Figure 18B), who was the Mayor of Póvoa de Varzim in the late 19th century. In addition, several other people also confirmed that he was in Porto on March 28.

Throughout the course of the trial, Vicente Urbino de Freitas maintained an extraordinary serenity, but as the prosecution witnesses were heard, his mood was gradually diminished. He began to lose the hope of being acquitted, such was the atmosphere of hatred and repulsion that surrounded upon him. Only his wife demonstrated an extraordinary dedication and was always by his side caressing him with her hands and words throughout the bitter hours. Vicente Urbino de Freitas was exhausted by the interrogation. As often happens with criminals, they hope to be safe from the responsibility of their crime, but a small error, a slightly suspicious circumstance, or reckless action committed in the blindness of the moment of the crime is enough to draw the attention of the prosecutors, open the judge’s eyes and point out the guilty party who attempted crimes against the life, honour or property of a fellow human being.

## Conclusion

The first judicial hearing took place on November 20, 1890. It was expected that it would be postponed due to the mourning period of Vicente Urbino de Freitas; on November 15, he had lost his 15-year-old daughter, who suffered from tuberculosis. However, the trial was not deferred. It was after 2 pm on December 1, 1893, but the court was crowded with curious spectators, all anxiously longing to know the final sentence of the thoroughly and minutely debated famous case. While the judge pronounced the sentence, an absolute silence reigned throughout the room. Indeed, the deafening silence made it possible to hear the convulsive sobbing of Vicente de Urbino de Freitas’ wife in the corner of the room, with her eyes tired from many sleepless nights. The judge read the following: “The public prosecutor accused Vicente Urbino de Freitas, a married man of 44 years, professor and doctor, natural of this city of Porto, of being the author of the crime of poisoning perpetrated upon the minor Mário Guilherme Augusto de Sampaio, in this city, of which he died in the afternoon of April 2, 1890. The jury found that the defendant is guilty of the crime of which he is accused and therefore he incurs in the penalty of article 353° of the penal code, which says that whoever commits a crime of poisoning will be punished with a prison sentence for 8 years, followed by deportation for 20 years, with a period of imprisonment of 2 years at the place of deportation (or without this, according to the opinion of the judge), or in the alternative with a fixed sentence of deportation for 28 years, in prison in the place of deportation for 8 to 10 years [1,3,11,13]. Since the jury found the aggravating circumstances of premeditation, of manifest superiority over the victim, by reason of age and family relationship in 3rd degree by civil law, and due to defendant’s insistence on the intent to commit the crime, and taking into account the attenuating circumstance of the defendant’s previous good conduct, I condemn the defendant to the sentence of 8 years in prison, followed by deportation for 20 years, without imprisonment in the place of deportation, or, alternatively, the penalty of deportation for 28 years with imprisonment for 8 years in the place of deportation. In either of the cases, the exile should occur in first class possession (i.e. in Portuguese colonies). Moreover, I condemn the defendant to the costs of the process.”

While hearing the sentence, Vicente Urbino de Freitas bowed his head and his wife fainted, which is one reason the judge ordered that she be removed from the room. Then, the judge addressed his words to the defendant: “According to the law, I have to make an exhortation to the defendant, but I do not know what kind of exhortation I can do to him. The defendant is sufficiently intelligent and learned. Therefore, I confine myself to telling him to resign himself to the penalty and to fulfil it with patience.” The trial was ended, and Vicente Urbino de Freitas was ordered to be taken to prison, which had already been his home for approximately 4 years. On the first days after the trial, he was very depressed and dejected and remained aloof and unavailable to the press, whom he had always dubbed to be unjust.

He regained tranquility and was visited by his wife and children, who showed him the greatest affection, every day. These visits were much like movie scenes in which the love and affection from his wife were most prominent. His imprisonment having weakened the income of his family (i.e. his wife and six children), Vicente Urbino de Freitas’ father-in-law had offered a subsistence pension to his daughter. Maria das Dores Basto Sampaio Freitas firmly rejected the subsistence pension, saying that her only family were her husband and children. Vicente Urbino de Freitas continued to proclaim his innocence, always assigning to a “double” the purchase and shipping of the package.

On a cold morning on May 27, 1894, at 6 am, Vicente Urbino de Freitas was awakened and made to follow the other convicts to the Lisbon Penitentiary. His wife, dedicated as always, was there with her husband once again, despite the terrible departure time, acting as his guardian angel. The guards approached the prisoners to handcuff them, joining the convicts together, and proceeding to walk them, two-by-two, to the train station. Vicente Urbino de Freitas protested loudly since he did not want to be linked to “bastards”. Once again, his wife, kneeling and holding the police officer’s hands, begged the man to allow her husband to go to the train station in a wagon drawn by horses escorted by four soldiers. The reports demonstrated that this whole ordeal was hideous and torturous for a man such as Vicente Urbino de Freitas, who lived in luxury and abundance and whom society had paid tribute to, showing respect and admiration.

Aiming to achieve the abovementioned conditions for her husband, his dedicated wife went to the police station to obtain permission for the escort, which was obtained. A crowd was there to see Vicente Urbino de Freitas at the prison exit and lined the streets along which he would pass, but a different route was followed. That night, he was admitted to the Limoeiro prison in Lisbon, and the following morning, May 28, 1894, he entered the Lisbon Penitentiary. His devoted wife continued to beg, pleading and crying at the feet of all who might alleviate her husband’s negative conditions within prison, and she eventually achieved her goal.

Vicente Urbino de Freitas, who chose to become the prison’s bookbinder (since in prison all prisoners were obliged to learn job), then enjoyed perks granted to no other prisoners during his stay. Due to prison regulations, he acquired other benefits and privileges provided in case of good behaviour. On certain days, he received visits from his family, who came to live in Lisbon.

He did not fulfil all 8 years of his prison sentence, as he took advantage of the commutation of his sentence granted by the royal pardon of May 12, 1898 as part of the commemoration of the centenary of the discovery of India [1–3,11,13,18]. He continued onto his exile in Angola on February 21, 1901 aboard the “Ambaca”. Following a new pardon by King D. Carlos in 1904, Vicente Urbino de Freitas went to Brazil and continued his medical practice, namely, dedicated to the treatment of his specialty, morphea, and accordingly news from the media seems to have made a new fortune. He must frequently have regretted his deviation from the path of honour and work to embark on the tortuous path of crime [16]. Indeed, Vicente Urbino de Freitas lived among the best of society and had an illustrious name in science, but his name became forever linked to the annals of monstrous crimes that justice was called to punish. Moreover, it should be noted that the first trial resulted in a hung jury. Thus, the lack of unanimity shows that some jurors were not convinced of his guilt [12]. Despite the strong judicial evidence, doubts still remain as to whether the “monster” was indeed a victim of circumstances and a hapless martyr [18,20].

His proven alibis testified that Vicente Urbino de Freitas was in Porto on March 27 and 28, but the testimonies of the Pucci (i.e. the confectioner from Capelistas Street) and the Gil stationery receptionist from Augusta Street, who both recognized the “ghost presence” of Vicente Urbino de Freitas in their shops on March 28, attest to the fragility of the testimonial evidence. Other receptionists also claimed that the purchase happened in their stores and others claimed that the package was sent by mail from their stores i.e., a torrent of contradictory testimonials). Although Vicente Urbino de Freitas had always denied any involvement in the crime, the evidence presented against him, though totally circumstantial, did compromise him completely [1–3,11,13,18].

Analysing the facts carefully, it appears that the main witness of the accusation was Vicente Urbino de Freitas himself. Indeed, in concealing very relevant facts (*e.g.*, the administration of the clysters, asking in a letter for Francisco Adolfo Coelho to forge a false alibi) and having been proved to have lied, Vicente Urbino de Freitas accused himself. Why? Self-hatred? It is impossible to determine. Vicente Urbino de Freitas had always acknowledged the existence of poisoning and never changed his conviction during the interrogations to which he was subjected. Literature also shows us that, with some exceptions, most poisonings are perpetrated by people with deep knowledge who attempt to leave no trace. Therefore, toxicological analyses would certainly have been decisive. In the next and last paper, all the forensic toxicological evidence will be analysed in light of 21st century knowledge. It is expected that we will come to understand how this evidence could be important in the outcome of this case. From the point of view of the testimonial evidence, everything points to Vicente Urbino de Freitas being guilty. By using Forensic Toxicology, we will see how the story ends. It will certainly make a good movie.
